# Supplementary material for: Genome-wide identification, characterization and gene expression of BES1 transcription factor family in grapevine (Vitis vinifera L.)
Source: Sci Rep. 2023 Jan 5;13:240. doi: 10.1038/s41598-022-24407-y (PMC9816167; doi:10.1038/s41598-022-24407-y)
Supplement: Supplementary file 3 — Supplementary Information. [file 41598_2022_24407_MOESM3_ESM.zip › Vvi_Atr/Vitis_vinifera.PN40024.v4.dna_sm.toplevel.fa.vs.Amborella_trichopoda.AMTR1.0.dna_sm.toplevel.fa.html/Vvi-17.html]

|  |  |  |  |  |  |  |  |  |  |  |  |  |  |
| --- | --- | --- | --- | --- | --- | --- | --- | --- | --- | --- | --- | --- | --- |
| Duplication depth | Reference chromosome | Collinear blocks | | | | | | | | | | | |
| 0 | Vvi-Vitvi17g04000\_t001 |  |  |  |  |  |  |  |  |  |  |  |  |
| 0 | Vvi-Vitvi17g00001\_t001 |  |  |  |  |  |  |  |  |  |  |  |  |
| 0 | Vvi-Vitvi17g00002\_t001 |  |  |  |  |  |  |  |  |  |  |  |  |
| 0 | Vvi-Vitvi17g01311\_t001 |  |  |  |  |  |  |  |  |  |  |  |  |
| 0 | Vvi-Vitvi17g00003\_t001 |  |  |  |  |  |  |  |  |  |  |  |  |
| 0 | Vvi-Vitvi17g00004\_t002 |  |  |  |  |  |  |  |  |  |  |  |  |
| 0 | Vvi-Vitvi17g00005\_t001 |  |  |  |  |  |  |  |  |  |  |  |  |
| 0 | Vvi-Vitvi17g04001\_t001 |  |  |  |  |  |  |  |  |  |  |  |  |
| 0 | Vvi-Vitvi17g04002\_t001 |  |  |  |  |  |  |  |  |  |  |  |  |
| 0 | Vvi-Vitvi17g04003\_t001 |  |  |  |  |  |  |  |  |  |  |  |  |
| 0 | Vvi-Vitvi17g04004\_t001 |  |  |  |  |  |  |  |  |  |  |  |  |
| 0 | Vvi-Vitvi17g00011\_t001 |  |  |  |  |  |  |  |  |  |  |  |  |
| 0 | Vvi-Vitvi17g00012\_t001 |  |  |  |  |  |  |  |  |  |  |  |  |
| 0 | Vvi-Vitvi17g00014\_t001 |  |  |  |  |  |  |  |  |  |  |  |  |
| 0 | Vvi-Vitvi17g01318\_t001 |  |  |  |  |  |  |  |  |  |  |  |  |
| 0 | Vvi-Vitvi17g01319\_t001 |  |  |  |  |  |  |  |  |  |  |  |  |
| 0 | Vvi-Vitvi17g00015\_t001 |  |  |  |  |  |  |  |  |  |  |  |  |
| 1 | Vvi-Vitvi17g00016\_t001 |  | Atr-ERN06124 |  |  |  |  |  |  |  |  |  |  |
| 1 | Vvi-Vitvi17g00017\_t001 |  | Atr-ERN06128 |  |  |  |  |  |  |  |  |  |  |
| 1 | Vvi-Vitvi17g00018\_t001 |  | Atr-ERN06130 |  |  |  |  |  |  |  |  |  |  |
| 1 | Vvi-Vitvi17g00019\_t002 |  | \| \| |  |  |  |  |  |  |  |  |  |  |
| 1 | Vvi-Vitvi17g04005\_t001 |  | \| \| |  |  |  |  |  |  |  |  |  |  |
| 1 | Vvi-Vitvi17g00020\_t001 |  | \| \| |  |  |  |  |  |  |  |  |  |  |
| 1 | Vvi-Vitvi17g04006\_t001 |  | \| \| |  |  |  |  |  |  |  |  |  |  |
| 1 | Vvi-Vitvi17g00021\_t001 |  | Atr-ERN06132 |  |  |  |  |  |  |  |  |  |  |
| 1 | Vvi-Vitvi17g00022\_t001 |  | \| \| |  |  |  |  |  |  |  |  |  |  |
| 1 | Vvi-Vitvi17g01320\_t001 |  | \| \| |  |  |  |  |  |  |  |  |  |  |
| 1 | Vvi-Vitvi17g04007\_t001 |  | Atr-ERN06135 |  |  |  |  |  |  |  |  |  |  |
| 1 | Vvi-Vitvi17g00024\_t001 |  | \| \| |  |  |  |  |  |  |  |  |  |  |
| 1 | Vvi-Vitvi17g00025\_t001 |  | Atr-ERN06140 |  |  |  |  |  |  |  |  |  |  |
| 1 | Vvi-Vitvi17g00026\_t001 |  | \| \| |  |  |  |  |  |  |  |  |  |  |
| 1 | Vvi-Vitvi17g00027\_t002 |  | \| \| |  |  |  |  |  |  |  |  |  |  |
| 1 | Vvi-Vitvi17g00028\_t001 |  | Atr-ERN06149 |  |  |  |  |  |  |  |  |  |  |
| 1 | Vvi-Vitvi17g01322\_t001 |  | \| \| |  |  |  |  |  |  |  |  |  |  |
| 1 | Vvi-Vitvi17g00029\_t001 |  | Atr-ERN06152 |  |  |  |  |  |  |  |  |  |  |
| 1 | Vvi-Vitvi17g00030\_t001 |  | \| \| |  |  |  |  |  |  |  |  |  |  |
| 1 | Vvi-Vitvi17g00031\_t001 |  | Atr-ERN06155 |  |  |  |  |  |  |  |  |  |  |
| 0 | Vvi-Vitvi17g00032\_t001 |  |  |  |  |  |  |  |  |  |  |  |  |
| 1 | Vvi-Vitvi17g00033\_t001 |  | Atr-ERN06210 |  |  |  |  |  |  |  |  |  |  |
| 1 | Vvi-Vitvi17g00036\_t001 |  | Atr-ERN06215 |  |  |  |  |  |  |  |  |  |  |
| 1 | Vvi-Vitvi17g00037\_t001 |  | Atr-ERN06217 |  |  |  |  |  |  |  |  |  |  |
| 1 | Vvi-Vitvi17g00038\_t001 |  | \| \| |  |  |  |  |  |  |  |  |  |  |
| 1 | Vvi-Vitvi17g00039\_t001 |  | \| \| |  |  |  |  |  |  |  |  |  |  |
| 1 | Vvi-Vitvi17g00040\_t001 |  | \| \| |  |  |  |  |  |  |  |  |  |  |
| 1 | Vvi-Vitvi17g00041\_t001 |  | \| \| |  |  |  |  |  |  |  |  |  |  |
| 1 | Vvi-Vitvi17g04008\_t001 |  | \| \| |  |  |  |  |  |  |  |  |  |  |
| 1 | Vvi-Vitvi17g00044\_t001 |  | \| \| |  |  |  |  |  |  |  |  |  |  |
| 1 | Vvi-Vitvi17g00045\_t001 |  | \| \| |  |  |  |  |  |  |  |  |  |  |
| 1 | Vvi-Vitvi17g00046\_t001 |  | \| \| |  |  |  |  |  |  |  |  |  |  |
| 1 | Vvi-Vitvi17g04009\_t001 |  | \| \| |  |  |  |  |  |  |  |  |  |  |
| 1 | Vvi-Vitvi17g00048\_t001 |  | \| \| |  |  |  |  |  |  |  |  |  |  |
| 1 | Vvi-Vitvi17g00050\_t001 |  | \| \| |  |  |  |  |  |  |  |  |  |  |
| 1 | Vvi-Vitvi17g00051\_t001 |  | \| \| |  |  |  |  |  |  |  |  |  |  |
| 1 | Vvi-Vitvi17g01324\_t001 |  | \| \| |  |  |  |  |  |  |  |  |  |  |
| 1 | Vvi-Vitvi17g00052\_t001 |  | Atr-ERN06219 |  |  |  |  |  |  |  |  |  |  |
| 1 | Vvi-Vitvi17g00053\_t001 |  | Atr-ERN06223 |  |  |  |  |  |  |  |  |  |  |
| 1 | Vvi-Vitvi17g04010\_t001 |  | \| \| |  |  |  |  |  |  |  |  |  |  |
| 1 | Vvi-Vitvi17g00054\_t001 |  | Atr-ERN06234 |  |  |  |  |  |  |  |  |  |  |
| 1 | Vvi-Vitvi17g00056\_t001 |  | Atr-ERN06235 |  |  |  |  |  |  |  |  |  |  |
| 1 | Vvi-Vitvi17g04011\_t001 |  | \| \| |  |  |  |  |  |  |  |  |  |  |
| 1 | Vvi-Vitvi17g00057\_t001 |  | Atr-ERN06237 |  |  |  |  |  |  |  |  |  |  |
| 1 | Vvi-Vitvi17g01326\_t001 |  | \| \| |  |  |  |  |  |  |  |  |  |  |
| 1 | Vvi-Vitvi17g04012\_t001 |  | \| \| |  |  |  |  |  |  |  |  |  |  |
| 1 | Vvi-Vitvi17g00059\_t001 |  | \| \| |  |  |  |  |  |  |  |  |  |  |
| 1 | Vvi-Vitvi17g01328\_t001 |  | \| \| |  |  |  |  |  |  |  |  |  |  |
| 1 | Vvi-Vitvi17g00060\_t001 |  | Atr-ERN06239 |  |  |  |  |  |  |  |  |  |  |
| 1 | Vvi-Vitvi17g00061\_t001 |  | Atr-ERN06240 |  |  |  |  |  |  |  |  |  |  |
| 1 | Vvi-Vitvi17g00062\_t001 |  | \| \| |  |  |  |  |  |  |  |  |  |  |
| 1 | Vvi-Vitvi17g00064\_t001 |  | \| \| |  |  |  |  |  |  |  |  |  |  |
| 1 | Vvi-Vitvi17g00065\_t001 |  | \| \| |  |  |  |  |  |  |  |  |  |  |
| 1 | Vvi-Vitvi17g00066\_t001 |  | Atr-ERN06249 |  |  |  |  |  |  |  |  |  |  |
| 1 | Vvi-Vitvi17g00067\_t001 |  | Atr-ERN06251 |  |  |  |  |  |  |  |  |  |  |
| 1 | Vvi-Vitvi17g00068\_t001 |  | Atr-ERN06254 |  |  |  |  |  |  |  |  |  |  |
| 1 | Vvi-Vitvi17g00069\_t001 |  | Atr-ERN06256 |  |  |  |  |  |  |  |  |  |  |
| 1 | Vvi-Vitvi17g04013\_t001 |  | \| \| |  |  |  |  |  |  |  |  |  |  |
| 1 | Vvi-Vitvi17g00070\_t001 |  | \| \| |  |  |  |  |  |  |  |  |  |  |
| 1 | Vvi-Vitvi17g00071\_t001 |  | Atr-ERN06258 |  |  |  |  |  |  |  |  |  |  |
| 1 | Vvi-Vitvi17g01330\_t001 |  | \| \| |  |  |  |  |  |  |  |  |  |  |
| 1 | Vvi-Vitvi17g00072\_t001 |  | Atr-ERN06259 |  |  |  |  |  |  |  |  |  |  |
| 1 | Vvi-Vitvi17g00073\_t001 |  | Atr-ERN06262 |  |  |  |  |  |  |  |  |  |  |
| 1 | Vvi-Vitvi17g01331\_t001 |  | Atr-ERN06263 |  |  |  |  |  |  |  |  |  |  |
| 1 | Vvi-Vitvi17g00074\_t001 |  | Atr-ERN06267 |  |  |  |  |  |  |  |  |  |  |
| 1 | Vvi-Vitvi17g00075\_t001 |  | Atr-ERN06268 |  |  |  |  |  |  |  |  |  |  |
| 1 | Vvi-Vitvi17g00076\_t001 |  | Atr-ERN06269 |  |  |  |  |  |  |  |  |  |  |
| 1 | Vvi-Vitvi17g01333\_t001 |  | Atr-ERN06272 |  |  |  |  |  |  |  |  |  |  |
| 1 | Vvi-Vitvi17g01334\_t001 |  | \| \| |  |  |  |  |  |  |  |  |  |  |
| 1 | Vvi-Vitvi17g01335\_t001 |  | \| \| |  |  |  |  |  |  |  |  |  |  |
| 1 | Vvi-Vitvi17g00078\_t001 |  | Atr-ERN06274 |  |  |  |  |  |  |  |  |  |  |
| 1 | Vvi-Vitvi17g00079\_t001 |  | \| \| |  |  |  |  |  |  |  |  |  |  |
| 1 | Vvi-Vitvi17g00080\_t001 |  | Atr-ERN06282 |  |  |  |  |  |  |  |  |  |  |
| 1 | Vvi-Vitvi17g00081\_t002 |  | Atr-ERN06294 |  |  |  |  |  |  |  |  |  |  |
| 1 | Vvi-Vitvi17g00082\_t001 |  | Atr-ERN06295 |  |  |  |  |  |  |  |  |  |  |
| 1 | Vvi-Vitvi17g04014\_t001 |  | \| \| |  |  |  |  |  |  |  |  |  |  |
| 1 | Vvi-Vitvi17g00083\_t001 |  | Atr-ERN06301 |  |  |  |  |  |  |  |  |  |  |
| 1 | Vvi-Vitvi17g04015\_t001 |  | Atr-ERN06302 |  |  |  |  |  |  |  |  |  |  |
| 1 | Vvi-Vitvi17g00084\_t001 |  | Atr-ERN06304 |  |  |  |  |  |  |  |  |  |  |
| 1 | Vvi-Vitvi17g01338\_t001 |  | \| \| |  |  |  |  |  |  |  |  |  |  |
| 1 | Vvi-Vitvi17g00085\_t001 |  | Atr-ERN06314 |  |  |  |  |  |  |  |  |  |  |
| 0 | Vvi-Vitvi17g01339\_t001 |  |  |  |  |  |  |  |  |  |  |  |  |
| 0 | Vvi-Vitvi17g04016\_t001 |  |  |  |  |  |  |  |  |  |  |  |  |
| 0 | Vvi-Vitvi17g01340\_t001 |  |  |  |  |  |  |  |  |  |  |  |  |
| 0 | Vvi-Vitvi17g04017\_t001 |  |  |  |  |  |  |  |  |  |  |  |  |
| 1 | Vvi-Vitvi17g00087\_t001 |  | Atr-ERN09826 |  |  |  |  |  |  |  |  |  |  |
| 1 | Vvi-Vitvi17g01341\_t001 |  | \| \| |  |  |  |  |  |  |  |  |  |  |
| 1 | Vvi-Vitvi17g00088\_t001 |  | Atr-ERN09839 |  |  |  |  |  |  |  |  |  |  |
| 1 | Vvi-Vitvi17g04018\_t001 |  | Atr-ERN09840 |  |  |  |  |  |  |  |  |  |  |
| 1 | Vvi-Vitvi17g00090\_t002 |  | \| \| |  |  |  |  |  |  |  |  |  |  |
| 1 | Vvi-Vitvi17g00091\_t001 |  | Atr-ERN09843 |  |  |  |  |  |  |  |  |  |  |
| 1 | Vvi-Vitvi17g00093\_t001 |  | \| \| |  |  |  |  |  |  |  |  |  |  |
| 1 | Vvi-Vitvi17g00094\_t001 |  | \| \| |  |  |  |  |  |  |  |  |  |  |
| 1 | Vvi-Vitvi17g00095\_t001 |  | Atr-ERN09859 |  |  |  |  |  |  |  |  |  |  |
| 1 | Vvi-Vitvi17g00096\_t002 |  | Atr-ERN09863 |  |  |  |  |  |  |  |  |  |  |
| 1 | Vvi-Vitvi17g04019\_t001 |  | \| \| |  |  |  |  |  |  |  |  |  |  |
| 1 | Vvi-Vitvi17g00098\_t004 |  | Atr-ERN09882 |  |  |  |  |  |  |  |  |  |  |
| 1 | Vvi-Vitvi17g04020\_t001 |  | \| \| |  |  |  |  |  |  |  |  |  |  |
| 1 | Vvi-Vitvi17g00099\_t001 |  | \| \| |  |  |  |  |  |  |  |  |  |  |
| 1 | Vvi-Vitvi17g01345\_t001 |  | Atr-ERN09890 |  |  |  |  |  |  |  |  |  |  |
| 1 | Vvi-Vitvi17g00100\_t001 |  | Atr-ERN09897 |  |  |  |  |  |  |  |  |  |  |
| 1 | Vvi-Vitvi17g00101\_t001 |  | Atr-ERN09902 |  |  |  |  |  |  |  |  |  |  |
| 1 | Vvi-Vitvi17g04021\_t001 |  | \| \| |  |  |  |  |  |  |  |  |  |  |
| 1 | Vvi-Vitvi17g00102\_t001 |  | \| \| |  |  |  |  |  |  |  |  |  |  |
| 1 | Vvi-Vitvi17g00103\_t002 |  | Atr-ERN09908 |  |  |  |  |  |  |  |  |  |  |
| 1 | Vvi-Vitvi17g01346\_t001 |  | \| \| |  |  |  |  |  |  |  |  |  |  |
| 1 | Vvi-Vitvi17g00104\_t001 |  | \| \| |  |  |  |  |  |  |  |  |  |  |
| 1 | Vvi-Vitvi17g00105\_t001 |  | Atr-ERN09919 |  |  |  |  |  |  |  |  |  |  |
| 1 | Vvi-Vitvi17g00106\_t001 |  | \| \| |  |  |  |  |  |  |  |  |  |  |
| 1 | Vvi-Vitvi17g00108\_t001 |  | \| \| |  |  |  |  |  |  |  |  |  |  |
| 1 | Vvi-Vitvi17g04022\_t001 |  | \| \| |  |  |  |  |  |  |  |  |  |  |
| 1 | Vvi-Vitvi17g01347\_t001 |  | Atr-ERN09923 |  |  |  |  |  |  |  |  |  |  |
| 1 | Vvi-Vitvi17g00109\_t001 |  | Atr-ERN09928 |  |  |  |  |  |  |  |  |  |  |
| 1 | Vvi-Vitvi17g00110\_t001 |  | Atr-ERN09933 |  |  |  |  |  |  |  |  |  |  |
| 1 | Vvi-Vitvi17g00111\_t001 |  | \| \| |  |  |  |  |  |  |  |  |  |  |
| 1 | Vvi-Vitvi17g00112\_t001 |  | Atr-ERN09947 |  |  |  |  |  |  |  |  |  |  |
| 1 | Vvi-Vitvi17g01350\_t001 |  | Atr-ERN09951 |  |  |  |  |  |  |  |  |  |  |
| 1 | Vvi-Vitvi17g00113\_t001 |  | \| \| |  |  |  |  |  |  |  |  |  |  |
| 1 | Vvi-Vitvi17g00114\_t001 |  | \| \| |  |  |  |  |  |  |  |  |  |  |
| 1 | Vvi-Vitvi17g01351\_t001 |  | Atr-ERN09957 |  |  |  |  |  |  |  |  |  |  |
| 1 | Vvi-Vitvi17g00115\_t001 |  | \| \| |  |  |  |  |  |  |  |  |  |  |
| 1 | Vvi-Vitvi17g04023\_t001 |  | \| \| |  |  |  |  |  |  |  |  |  |  |
| 1 | Vvi-Vitvi17g00116\_t001 |  | Atr-ERN09962 |  |  |  |  |  |  |  |  |  |  |
| 1 | Vvi-Vitvi17g00118\_t001 |  | Atr-ERN09965 |  |  |  |  |  |  |  |  |  |  |
| 1 | Vvi-Vitvi17g00119\_t001 |  | Atr-ERN09972 |  |  |  |  |  |  |  |  |  |  |
| 1 | Vvi-Vitvi17g00120\_t001 |  | Atr-ERN09979 |  |  |  |  |  |  |  |  |  |  |
| 1 | Vvi-Vitvi17g00121\_t001 |  | Atr-ERN09981 |  |  |  |  |  |  |  |  |  |  |
| 1 | Vvi-Vitvi17g00122\_t001 |  | Atr-ERN09991 |  |  |  |  |  |  |  |  |  |  |
| 1 | Vvi-Vitvi17g00123\_t001 |  | \| \| |  |  |  |  |  |  |  |  |  |  |
| 1 | Vvi-Vitvi17g00124\_t002 |  | Atr-ERN10004 |  |  |  |  |  |  |  |  |  |  |
| 1 | Vvi-Vitvi17g00125\_t001 |  | Atr-ERN10008 |  |  |  |  |  |  |  |  |  |  |
| 1 | Vvi-Vitvi17g00127\_t001 |  | Atr-ERN10015 |  |  |  |  |  |  |  |  |  |  |
| 0 | Vvi-Vitvi17g04024\_t001 |  |  |  |  |  |  |  |  |  |  |  |  |
| 0 | Vvi-Vitvi17g00130\_t001 |  |  |  |  |  |  |  |  |  |  |  |  |
| 0 | Vvi-Vitvi17g00131\_t001 |  |  |  |  |  |  |  |  |  |  |  |  |
| 0 | Vvi-Vitvi17g00132\_t001 |  |  |  |  |  |  |  |  |  |  |  |  |
| 0 | Vvi-Vitvi17g00133\_t001 |  |  |  |  |  |  |  |  |  |  |  |  |
| 0 | Vvi-Vitvi17g00135\_t001 |  |  |  |  |  |  |  |  |  |  |  |  |
| 0 | Vvi-Vitvi17g00136\_t001 |  |  |  |  |  |  |  |  |  |  |  |  |
| 0 | Vvi-Vitvi17g00137\_t001 |  |  |  |  |  |  |  |  |  |  |  |  |
| 0 | Vvi-Vitvi17g00138\_t001 |  |  |  |  |  |  |  |  |  |  |  |  |
| 0 | Vvi-Vitvi17g00139\_t001 |  |  |  |  |  |  |  |  |  |  |  |  |
| 0 | Vvi-Vitvi17g00141\_t001 |  |  |  |  |  |  |  |  |  |  |  |  |
| 0 | Vvi-Vitvi17g00142\_t001 |  |  |  |  |  |  |  |  |  |  |  |  |
| 0 | Vvi-Vitvi17g00143\_t001 |  |  |  |  |  |  |  |  |  |  |  |  |
| 0 | Vvi-Vitvi17g00145\_t001 |  |  |  |  |  |  |  |  |  |  |  |  |
| 0 | Vvi-Vitvi17g04025\_t001 |  |  |  |  |  |  |  |  |  |  |  |  |
| 0 | Vvi-Vitvi17g04026\_t001 |  |  |  |  |  |  |  |  |  |  |  |  |
| 0 | Vvi-Vitvi17g01354\_t001 |  |  |  |  |  |  |  |  |  |  |  |  |
| 0 | Vvi-Vitvi17g00147\_t002 |  |  |  |  |  |  |  |  |  |  |  |  |
| 0 | Vvi-Vitvi17g04027\_t001 |  |  |  |  |  |  |  |  |  |  |  |  |
| 0 | Vvi-Vitvi17g04028\_t001 |  |  |  |  |  |  |  |  |  |  |  |  |
| 0 | Vvi-Vitvi17g00148\_t001 |  |  |  |  |  |  |  |  |  |  |  |  |
| 0 | Vvi-Vitvi17g00149\_t001 |  |  |  |  |  |  |  |  |  |  |  |  |
| 0 | Vvi-Vitvi17g04029\_t001 |  |  |  |  |  |  |  |  |  |  |  |  |
| 0 | Vvi-Vitvi17g00150\_t001 |  |  |  |  |  |  |  |  |  |  |  |  |
| 0 | Vvi-Vitvi17g01355\_t001 |  |  |  |  |  |  |  |  |  |  |  |  |
| 0 | Vvi-Vitvi17g00151\_t001 |  |  |  |  |  |  |  |  |  |  |  |  |
| 0 | Vvi-Vitvi17g00152\_t001 |  |  |  |  |  |  |  |  |  |  |  |  |
| 0 | Vvi-Vitvi17g00153\_t002 |  |  |  |  |  |  |  |  |  |  |  |  |
| 0 | Vvi-Vitvi17g00154\_t001 |  |  |  |  |  |  |  |  |  |  |  |  |
| 0 | Vvi-Vitvi17g00155\_t001 |  |  |  |  |  |  |  |  |  |  |  |  |
| 0 | Vvi-Vitvi17g00156\_t002 |  |  |  |  |  |  |  |  |  |  |  |  |
| 0 | Vvi-Vitvi17g04030\_t001 |  |  |  |  |  |  |  |  |  |  |  |  |
| 0 | Vvi-Vitvi17g00157\_t001 |  |  |  |  |  |  |  |  |  |  |  |  |
| 0 | Vvi-Vitvi17g00159\_t001 |  |  |  |  |  |  |  |  |  |  |  |  |
| 0 | Vvi-Vitvi17g04031\_t001 |  |  |  |  |  |  |  |  |  |  |  |  |
| 0 | Vvi-Vitvi17g00160\_t001 |  |  |  |  |  |  |  |  |  |  |  |  |
| 0 | Vvi-Vitvi17g04032\_t001 |  |  |  |  |  |  |  |  |  |  |  |  |
| 0 | Vvi-Vitvi17g00161\_t001 |  |  |  |  |  |  |  |  |  |  |  |  |
| 0 | Vvi-Vitvi17g00162\_t001 |  |  |  |  |  |  |  |  |  |  |  |  |
| 0 | Vvi-Vitvi17g01358\_t001 |  |  |  |  |  |  |  |  |  |  |  |  |
| 0 | Vvi-Vitvi17g00163\_t001 |  |  |  |  |  |  |  |  |  |  |  |  |
| 0 | Vvi-Vitvi17g00164\_t001 |  |  |  |  |  |  |  |  |  |  |  |  |
| 0 | Vvi-Vitvi17g00165\_t001 |  |  |  |  |  |  |  |  |  |  |  |  |
| 0 | Vvi-Vitvi17g00166\_t001 |  |  |  |  |  |  |  |  |  |  |  |  |
| 0 | Vvi-Vitvi17g00167\_t001 |  |  |  |  |  |  |  |  |  |  |  |  |
| 0 | Vvi-Vitvi17g00168\_t001 |  |  |  |  |  |  |  |  |  |  |  |  |
| 0 | Vvi-Vitvi17g00169\_t001 |  |  |  |  |  |  |  |  |  |  |  |  |
| 0 | Vvi-Vitvi17g00170\_t001 |  |  |  |  |  |  |  |  |  |  |  |  |
| 0 | Vvi-Vitvi17g00171\_t001 |  |  |  |  |  |  |  |  |  |  |  |  |
| 0 | Vvi-Vitvi17g00172\_t001 |  |  |  |  |  |  |  |  |  |  |  |  |
| 0 | Vvi-Vitvi17g00173\_t001 |  |  |  |  |  |  |  |  |  |  |  |  |
| 0 | Vvi-Vitvi17g00174\_t001 |  |  |  |  |  |  |  |  |  |  |  |  |
| 0 | Vvi-Vitvi17g04033\_t001 |  |  |  |  |  |  |  |  |  |  |  |  |
| 0 | Vvi-Vitvi17g00175\_t001 |  |  |  |  |  |  |  |  |  |  |  |  |
| 0 | Vvi-Vitvi17g04034\_t001 |  |  |  |  |  |  |  |  |  |  |  |  |
| 0 | Vvi-Vitvi17g01359\_t001 |  |  |  |  |  |  |  |  |  |  |  |  |
| 0 | Vvi-Vitvi17g01360\_t001 |  |  |  |  |  |  |  |  |  |  |  |  |
| 0 | Vvi-Vitvi17g00177\_t001 |  |  |  |  |  |  |  |  |  |  |  |  |
| 0 | Vvi-Vitvi17g04035\_t001 |  |  |  |  |  |  |  |  |  |  |  |  |
| 0 | Vvi-Vitvi17g00178\_t001 |  |  |  |  |  |  |  |  |  |  |  |  |
| 0 | Vvi-Vitvi17g00179\_t001 |  |  |  |  |  |  |  |  |  |  |  |  |
| 0 | Vvi-Vitvi17g00181\_t001 |  |  |  |  |  |  |  |  |  |  |  |  |
| 0 | Vvi-Vitvi17g00183\_t001 |  |  |  |  |  |  |  |  |  |  |  |  |
| 0 | Vvi-Vitvi17g00186\_t001 |  |  |  |  |  |  |  |  |  |  |  |  |
| 0 | Vvi-Vitvi17g01361\_t001 |  |  |  |  |  |  |  |  |  |  |  |  |
| 0 | Vvi-Vitvi17g00187\_t001 |  |  |  |  |  |  |  |  |  |  |  |  |
| 0 | Vvi-Vitvi17g00188\_t001 |  |  |  |  |  |  |  |  |  |  |  |  |
| 0 | Vvi-Vitvi17g00189\_t002 |  |  |  |  |  |  |  |  |  |  |  |  |
| 0 | Vvi-Vitvi17g04036\_t001 |  |  |  |  |  |  |  |  |  |  |  |  |
| 0 | Vvi-Vitvi17g00190\_t001 |  |  |  |  |  |  |  |  |  |  |  |  |
| 0 | Vvi-Vitvi17g04037\_t001 |  |  |  |  |  |  |  |  |  |  |  |  |
| 0 | Vvi-Vitvi17g00191\_t001 |  |  |  |  |  |  |  |  |  |  |  |  |
| 0 | Vvi-Vitvi17g04038\_t001 |  |  |  |  |  |  |  |  |  |  |  |  |
| 0 | Vvi-Vitvi17g00192\_t001 |  |  |  |  |  |  |  |  |  |  |  |  |
| 0 | Vvi-Vitvi17g00194\_t001 |  |  |  |  |  |  |  |  |  |  |  |  |
| 0 | Vvi-Vitvi17g00195\_t001 |  |  |  |  |  |  |  |  |  |  |  |  |
| 0 | Vvi-Vitvi17g04039\_t001 |  |  |  |  |  |  |  |  |  |  |  |  |
| 0 | Vvi-Vitvi17g00196\_t002 |  |  |  |  |  |  |  |  |  |  |  |  |
| 0 | Vvi-Vitvi17g00197\_t001 |  |  |  |  |  |  |  |  |  |  |  |  |
| 0 | Vvi-Vitvi17g00198\_t001 |  |  |  |  |  |  |  |  |  |  |  |  |
| 0 | Vvi-Vitvi17g00199\_t001 |  |  |  |  |  |  |  |  |  |  |  |  |
| 0 | Vvi-Vitvi17g00200\_t001 |  |  |  |  |  |  |  |  |  |  |  |  |
| 0 | Vvi-Vitvi17g01362\_t001 |  |  |  |  |  |  |  |  |  |  |  |  |
| 0 | Vvi-Vitvi17g04040\_t001 |  |  |  |  |  |  |  |  |  |  |  |  |
| 0 | Vvi-Vitvi17g04041\_t001 |  |  |  |  |  |  |  |  |  |  |  |  |
| 0 | Vvi-Vitvi17g04042\_t001 |  |  |  |  |  |  |  |  |  |  |  |  |
| 0 | Vvi-Vitvi17g01365\_t001 |  |  |  |  |  |  |  |  |  |  |  |  |
| 0 | Vvi-Vitvi17g00203\_t001 |  |  |  |  |  |  |  |  |  |  |  |  |
| 0 | Vvi-Vitvi17g04043\_t001 |  |  |  |  |  |  |  |  |  |  |  |  |
| 0 | Vvi-Vitvi17g04044\_t001 |  |  |  |  |  |  |  |  |  |  |  |  |
| 0 | Vvi-Vitvi17g00205\_t001 |  |  |  |  |  |  |  |  |  |  |  |  |
| 0 | Vvi-Vitvi17g00206\_t001 |  |  |  |  |  |  |  |  |  |  |  |  |
| 0 | Vvi-Vitvi17g04045\_t001 |  |  |  |  |  |  |  |  |  |  |  |  |
| 0 | Vvi-Vitvi17g04046\_t001 |  |  |  |  |  |  |  |  |  |  |  |  |
| 0 | Vvi-Vitvi17g00209\_t001 |  |  |  |  |  |  |  |  |  |  |  |  |
| 0 | Vvi-Vitvi17g00210\_t001 |  |  |  |  |  |  |  |  |  |  |  |  |
| 0 | Vvi-Vitvi17g00213\_t001 |  |  |  |  |  |  |  |  |  |  |  |  |
| 0 | Vvi-Vitvi17g01368\_t001 |  |  |  |  |  |  |  |  |  |  |  |  |
| 0 | Vvi-Vitvi17g00214\_t001 |  |  |  |  |  |  |  |  |  |  |  |  |
| 0 | Vvi-Vitvi17g04047\_t001 |  |  |  |  |  |  |  |  |  |  |  |  |
| 0 | Vvi-Vitvi17g04048\_t001 |  |  |  |  |  |  |  |  |  |  |  |  |
| 0 | Vvi-Vitvi17g04049\_t001 |  |  |  |  |  |  |  |  |  |  |  |  |
| 0 | Vvi-Vitvi17g04050\_t001 |  |  |  |  |  |  |  |  |  |  |  |  |
| 0 | Vvi-Vitvi17g04051\_t001 |  |  |  |  |  |  |  |  |  |  |  |  |
| 0 | Vvi-Vitvi17g00216\_t001 |  |  |  |  |  |  |  |  |  |  |  |  |
| 0 | Vvi-Vitvi17g00217\_t001 |  |  |  |  |  |  |  |  |  |  |  |  |
| 0 | Vvi-Vitvi17g00218\_t001 |  |  |  |  |  |  |  |  |  |  |  |  |
| 0 | Vvi-Vitvi17g00219\_t001 |  |  |  |  |  |  |  |  |  |  |  |  |
| 0 | Vvi-Vitvi17g00220\_t001 |  |  |  |  |  |  |  |  |  |  |  |  |
| 0 | Vvi-Vitvi17g04052\_t001 |  |  |  |  |  |  |  |  |  |  |  |  |
| 0 | Vvi-Vitvi17g01370\_t001 |  |  |  |  |  |  |  |  |  |  |  |  |
| 0 | Vvi-Vitvi17g00222\_t001 |  |  |  |  |  |  |  |  |  |  |  |  |
| 0 | Vvi-Vitvi17g04053\_t001 |  |  |  |  |  |  |  |  |  |  |  |  |
| 0 | Vvi-Vitvi17g00223\_t001 |  |  |  |  |  |  |  |  |  |  |  |  |
| 0 | Vvi-Vitvi17g04054\_t001 |  |  |  |  |  |  |  |  |  |  |  |  |
| 0 | Vvi-Vitvi17g00225\_t001 |  |  |  |  |  |  |  |  |  |  |  |  |
| 0 | Vvi-Vitvi17g00226\_t001 |  |  |  |  |  |  |  |  |  |  |  |  |
| 0 | Vvi-Vitvi17g00227\_t001 |  |  |  |  |  |  |  |  |  |  |  |  |
| 0 | Vvi-Vitvi17g00228\_t001 |  |  |  |  |  |  |  |  |  |  |  |  |
| 0 | Vvi-Vitvi17g01371\_t001 |  |  |  |  |  |  |  |  |  |  |  |  |
| 0 | Vvi-Vitvi17g00229\_t001 |  |  |  |  |  |  |  |  |  |  |  |  |
| 0 | Vvi-Vitvi17g04055\_t001 |  |  |  |  |  |  |  |  |  |  |  |  |
| 0 | Vvi-Vitvi17g00230\_t001 |  |  |  |  |  |  |  |  |  |  |  |  |
| 0 | Vvi-Vitvi17g00231\_t001 |  |  |  |  |  |  |  |  |  |  |  |  |
| 0 | Vvi-Vitvi17g00232\_t001 |  |  |  |  |  |  |  |  |  |  |  |  |
| 0 | Vvi-Vitvi17g04056\_t001 |  |  |  |  |  |  |  |  |  |  |  |  |
| 0 | Vvi-Vitvi17g00233\_t001 |  |  |  |  |  |  |  |  |  |  |  |  |
| 0 | Vvi-Vitvi17g00234\_t001 |  |  |  |  |  |  |  |  |  |  |  |  |
| 0 | Vvi-Vitvi17g00235\_t001 |  |  |  |  |  |  |  |  |  |  |  |  |
| 0 | Vvi-Vitvi17g00237\_t001 |  |  |  |  |  |  |  |  |  |  |  |  |
| 0 | Vvi-Vitvi17g00238\_t001 |  |  |  |  |  |  |  |  |  |  |  |  |
| 0 | Vvi-Vitvi17g00239\_t001 |  |  |  |  |  |  |  |  |  |  |  |  |
| 0 | Vvi-Vitvi17g00240\_t001 |  |  |  |  |  |  |  |  |  |  |  |  |
| 0 | Vvi-Vitvi17g01373\_t001 |  |  |  |  |  |  |  |  |  |  |  |  |
| 0 | Vvi-Vitvi17g01374\_t001 |  |  |  |  |  |  |  |  |  |  |  |  |
| 0 | Vvi-Vitvi17g04057\_t001 |  |  |  |  |  |  |  |  |  |  |  |  |
| 0 | Vvi-Vitvi17g00241\_t001 |  |  |  |  |  |  |  |  |  |  |  |  |
| 0 | Vvi-Vitvi17g04058\_t001 |  |  |  |  |  |  |  |  |  |  |  |  |
| 0 | Vvi-Vitvi17g04059\_t001 |  |  |  |  |  |  |  |  |  |  |  |  |
| 0 | Vvi-Vitvi17g04060\_t001 |  |  |  |  |  |  |  |  |  |  |  |  |
| 0 | Vvi-Vitvi17g00245\_t001 |  |  |  |  |  |  |  |  |  |  |  |  |
| 0 | Vvi-Vitvi17g00246\_t001 |  |  |  |  |  |  |  |  |  |  |  |  |
| 0 | Vvi-Vitvi17g01376\_t001 |  |  |  |  |  |  |  |  |  |  |  |  |
| 0 | Vvi-Vitvi17g00247\_t001 |  |  |  |  |  |  |  |  |  |  |  |  |
| 0 | Vvi-Vitvi17g00250\_t001 |  |  |  |  |  |  |  |  |  |  |  |  |
| 0 | Vvi-Vitvi17g04061\_t001 |  |  |  |  |  |  |  |  |  |  |  |  |
| 0 | Vvi-Vitvi17g01377\_t001 |  |  |  |  |  |  |  |  |  |  |  |  |
| 0 | Vvi-Vitvi17g01378\_t001 |  |  |  |  |  |  |  |  |  |  |  |  |
| 0 | Vvi-Vitvi17g00251\_t001 |  |  |  |  |  |  |  |  |  |  |  |  |
| 0 | Vvi-Vitvi17g04062\_t001 |  |  |  |  |  |  |  |  |  |  |  |  |
| 0 | Vvi-Vitvi17g00252\_t001 |  |  |  |  |  |  |  |  |  |  |  |  |
| 0 | Vvi-Vitvi17g00253\_t001 |  |  |  |  |  |  |  |  |  |  |  |  |
| 0 | Vvi-Vitvi17g00254\_t001 |  |  |  |  |  |  |  |  |  |  |  |  |
| 0 | Vvi-Vitvi17g01379\_t001 |  |  |  |  |  |  |  |  |  |  |  |  |
| 0 | Vvi-Vitvi17g01380\_t001 |  |  |  |  |  |  |  |  |  |  |  |  |
| 0 | Vvi-Vitvi17g01381\_t001 |  |  |  |  |  |  |  |  |  |  |  |  |
| 0 | Vvi-Vitvi17g01382\_t001 |  |  |  |  |  |  |  |  |  |  |  |  |
| 0 | Vvi-Vitvi17g00255\_t001 |  |  |  |  |  |  |  |  |  |  |  |  |
| 0 | Vvi-Vitvi17g00258\_t002 |  |  |  |  |  |  |  |  |  |  |  |  |
| 0 | Vvi-Vitvi17g00257\_t001 |  |  |  |  |  |  |  |  |  |  |  |  |
| 0 | Vvi-Vitvi17g00259\_t001 |  |  |  |  |  |  |  |  |  |  |  |  |
| 0 | Vvi-Vitvi17g00260\_t001 |  |  |  |  |  |  |  |  |  |  |  |  |
| 0 | Vvi-Vitvi17g00261\_t001 |  |  |  |  |  |  |  |  |  |  |  |  |
| 0 | Vvi-Vitvi17g00262\_t001 |  |  |  |  |  |  |  |  |  |  |  |  |
| 0 | Vvi-Vitvi17g00263\_t001 |  |  |  |  |  |  |  |  |  |  |  |  |
| 0 | Vvi-Vitvi17g00264\_t001 |  |  |  |  |  |  |  |  |  |  |  |  |
| 0 | Vvi-Vitvi17g04063\_t001 |  |  |  |  |  |  |  |  |  |  |  |  |
| 0 | Vvi-Vitvi17g04064\_t001 |  |  |  |  |  |  |  |  |  |  |  |  |
| 0 | Vvi-Vitvi17g00265\_t001 |  |  |  |  |  |  |  |  |  |  |  |  |
| 0 | Vvi-Vitvi17g04065\_t001 |  |  |  |  |  |  |  |  |  |  |  |  |
| 0 | Vvi-Vitvi17g04066\_t001 |  |  |  |  |  |  |  |  |  |  |  |  |
| 0 | Vvi-Vitvi17g04067\_t001 |  |  |  |  |  |  |  |  |  |  |  |  |
| 0 | Vvi-Vitvi17g04068\_t001 |  |  |  |  |  |  |  |  |  |  |  |  |
| 0 | Vvi-Vitvi17g01386\_t001 |  |  |  |  |  |  |  |  |  |  |  |  |
| 0 | Vvi-Vitvi17g00268\_t001 |  |  |  |  |  |  |  |  |  |  |  |  |
| 0 | Vvi-Vitvi17g04069\_t001 |  |  |  |  |  |  |  |  |  |  |  |  |
| 0 | Vvi-Vitvi17g00270\_t001 |  |  |  |  |  |  |  |  |  |  |  |  |
| 0 | Vvi-Vitvi17g04070\_t001 |  |  |  |  |  |  |  |  |  |  |  |  |
| 0 | Vvi-Vitvi17g00271\_t001 |  |  |  |  |  |  |  |  |  |  |  |  |
| 0 | Vvi-Vitvi17g04071\_t001 |  |  |  |  |  |  |  |  |  |  |  |  |
| 0 | Vvi-Vitvi17g00273\_t001 |  |  |  |  |  |  |  |  |  |  |  |  |
| 0 | Vvi-Vitvi17g00274\_t001 |  |  |  |  |  |  |  |  |  |  |  |  |
| 0 | Vvi-Vitvi17g00275\_t001 |  |  |  |  |  |  |  |  |  |  |  |  |
| 0 | Vvi-Vitvi17g04072\_t001 |  |  |  |  |  |  |  |  |  |  |  |  |
| 0 | Vvi-Vitvi17g04073\_t001 |  |  |  |  |  |  |  |  |  |  |  |  |
| 0 | Vvi-Vitvi17g04074\_t001 |  |  |  |  |  |  |  |  |  |  |  |  |
| 0 | Vvi-Vitvi17g00277\_t001 |  |  |  |  |  |  |  |  |  |  |  |  |
| 0 | Vvi-Vitvi17g00278\_t001 |  |  |  |  |  |  |  |  |  |  |  |  |
| 0 | Vvi-Vitvi17g04075\_t001 |  |  |  |  |  |  |  |  |  |  |  |  |
| 0 | Vvi-Vitvi17g00279\_t001 |  |  |  |  |  |  |  |  |  |  |  |  |
| 0 | Vvi-Vitvi17g00280\_t001 |  |  |  |  |  |  |  |  |  |  |  |  |
| 0 | Vvi-Vitvi17g00281\_t001 |  |  |  |  |  |  |  |  |  |  |  |  |
| 0 | Vvi-Vitvi17g04076\_t001 |  |  |  |  |  |  |  |  |  |  |  |  |
| 0 | Vvi-Vitvi17g00282\_t001 |  |  |  |  |  |  |  |  |  |  |  |  |
| 0 | Vvi-Vitvi17g01391\_t001 |  |  |  |  |  |  |  |  |  |  |  |  |
| 0 | Vvi-Vitvi17g00283\_t001 |  |  |  |  |  |  |  |  |  |  |  |  |
| 1 | Vvi-Vitvi17g00284\_t001 |  | Atr-ERN04050 |  |  |  |  |  |  |  |  |  |  |
| 1 | Vvi-Vitvi17g00285\_t001 |  | \| \| |  |  |  |  |  |  |  |  |  |  |
| 1 | Vvi-Vitvi17g00287\_t001 |  | \| \| |  |  |  |  |  |  |  |  |  |  |
| 1 | Vvi-Vitvi17g04077\_t001 |  | \| \| |  |  |  |  |  |  |  |  |  |  |
| 1 | Vvi-Vitvi17g01392\_t001 |  | Atr-ERN04046 |  |  |  |  |  |  |  |  |  |  |
| 1 | Vvi-Vitvi17g04078\_t001 |  | \| \| |  |  |  |  |  |  |  |  |  |  |
| 1 | Vvi-Vitvi17g00289\_t001 |  | Atr-ERN04045 |  |  |  |  |  |  |  |  |  |  |
| 1 | Vvi-Vitvi17g01393\_t001 |  | \| \| |  |  |  |  |  |  |  |  |  |  |
| 1 | Vvi-Vitvi17g01394\_t001 |  | \| \| |  |  |  |  |  |  |  |  |  |  |
| 1 | Vvi-Vitvi17g01395\_t001 |  | \| \| |  |  |  |  |  |  |  |  |  |  |
| 1 | Vvi-Vitvi17g04079\_t001 |  | \| \| |  |  |  |  |  |  |  |  |  |  |
| 1 | Vvi-Vitvi17g04080\_t001 |  | \| \| |  |  |  |  |  |  |  |  |  |  |
| 1 | Vvi-Vitvi17g01396\_t001 |  | \| \| |  |  |  |  |  |  |  |  |  |  |
| 1 | Vvi-Vitvi17g00290\_t001 |  | \| \| |  |  |  |  |  |  |  |  |  |  |
| 1 | Vvi-Vitvi17g00291\_t001 |  | \| \| |  |  |  |  |  |  |  |  |  |  |
| 1 | Vvi-Vitvi17g00292\_t001 |  | \| \| |  |  |  |  |  |  |  |  |  |  |
| 1 | Vvi-Vitvi17g00293\_t001 |  | \| \| |  |  |  |  |  |  |  |  |  |  |
| 1 | Vvi-Vitvi17g00294\_t001 |  | Atr-ERN04031 |  |  |  |  |  |  |  |  |  |  |
| 1 | Vvi-Vitvi17g00295\_t001 |  | \| \| |  |  |  |  |  |  |  |  |  |  |
| 1 | Vvi-Vitvi17g00296\_t001 |  | \| \| |  |  |  |  |  |  |  |  |  |  |
| 1 | Vvi-Vitvi17g04081\_t001 |  | \| \| |  |  |  |  |  |  |  |  |  |  |
| 1 | Vvi-Vitvi17g00298\_t001 |  | Atr-ERN04027 |  |  |  |  |  |  |  |  |  |  |
| 1 | Vvi-Vitvi17g00299\_t001 |  | Atr-ERN04022 |  |  |  |  |  |  |  |  |  |  |
| 1 | Vvi-Vitvi17g04082\_t001 |  | Atr-ERN04006 |  |  |  |  |  |  |  |  |  |  |
| 1 | Vvi-Vitvi17g00300\_t001 |  | Atr-ERN04001 |  |  |  |  |  |  |  |  |  |  |
| 1 | Vvi-Vitvi17g00301\_t001 |  | Atr-ERN04000 |  |  |  |  |  |  |  |  |  |  |
| 1 | Vvi-Vitvi17g01399\_t001 |  | \| \| |  |  |  |  |  |  |  |  |  |  |
| 1 | Vvi-Vitvi17g00302\_t001 |  | \| \| |  |  |  |  |  |  |  |  |  |  |
| 1 | Vvi-Vitvi17g00303\_t001 |  | Atr-ERN03998 |  |  |  |  |  |  |  |  |  |  |
| 1 | Vvi-Vitvi17g01400\_t001 |  | \| \| |  |  |  |  |  |  |  |  |  |  |
| 1 | Vvi-Vitvi17g00304\_t001 |  | Atr-ERN03996 |  |  |  |  |  |  |  |  |  |  |
| 1 | Vvi-Vitvi17g00305\_t001 |  | \| \| |  |  |  |  |  |  |  |  |  |  |
| 1 | Vvi-Vitvi17g00306\_t001 |  | \| \| |  |  |  |  |  |  |  |  |  |  |
| 1 | Vvi-Vitvi17g00307\_t001 |  | \| \| |  |  |  |  |  |  |  |  |  |  |
| 1 | Vvi-Vitvi17g00309\_t001 |  | Atr-ERN03991 |  |  |  |  |  |  |  |  |  |  |
| 1 | Vvi-Vitvi17g01401\_t001 |  | \| \| |  |  |  |  |  |  |  |  |  |  |
| 1 | Vvi-Vitvi17g04083\_t001 |  | \| \| |  |  |  |  |  |  |  |  |  |  |
| 1 | Vvi-Vitvi17g00311\_t001 |  | Atr-ERN03989 |  |  |  |  |  |  |  |  |  |  |
| 1 | Vvi-Vitvi17g00312\_t002 |  | Atr-ERN03983 |  |  |  |  |  |  |  |  |  |  |
| 1 | Vvi-Vitvi17g01402\_t001 |  | Atr-ERN03981 |  |  |  |  |  |  |  |  |  |  |
| 1 | Vvi-Vitvi17g00313\_t001 |  | Atr-ERN03977 |  |  |  |  |  |  |  |  |  |  |
| 1 | Vvi-Vitvi17g00314\_t001 |  | \| \| |  |  |  |  |  |  |  |  |  |  |
| 1 | Vvi-Vitvi17g01403\_t001 |  | \| \| |  |  |  |  |  |  |  |  |  |  |
| 1 | Vvi-Vitvi17g00315\_t001 |  | Atr-ERN03974 |  |  |  |  |  |  |  |  |  |  |
| 1 | Vvi-Vitvi17g01404\_t001 |  | \| \| |  |  |  |  |  |  |  |  |  |  |
| 1 | Vvi-Vitvi17g01405\_t001 |  | \| \| |  |  |  |  |  |  |  |  |  |  |
| 1 | Vvi-Vitvi17g00316\_t001 |  | \| \| |  |  |  |  |  |  |  |  |  |  |
| 1 | Vvi-Vitvi17g04084\_t001 |  | \| \| |  |  |  |  |  |  |  |  |  |  |
| 1 | Vvi-Vitvi17g00318\_t001 |  | Atr-ERN03967 |  |  |  |  |  |  |  |  |  |  |
| 1 | Vvi-Vitvi17g04085\_t001 |  | \| \| |  |  |  |  |  |  |  |  |  |  |
| 1 | Vvi-Vitvi17g00320\_t001 |  | Atr-ERN03952 |  |  |  |  |  |  |  |  |  |  |
| 1 | Vvi-Vitvi17g00321\_t001 |  | \| \| |  |  |  |  |  |  |  |  |  |  |
| 1 | Vvi-Vitvi17g00324\_t001 |  | \| \| |  |  |  |  |  |  |  |  |  |  |
| 1 | Vvi-Vitvi17g00325\_t001 |  | \| \| |  |  |  |  |  |  |  |  |  |  |
| 1 | Vvi-Vitvi17g04086\_t001 |  | \| \| |  |  |  |  |  |  |  |  |  |  |
| 1 | Vvi-Vitvi17g00326\_t001 |  | \| \| |  |  |  |  |  |  |  |  |  |  |
| 1 | Vvi-Vitvi17g00327\_t001 |  | \| \| |  |  |  |  |  |  |  |  |  |  |
| 1 | Vvi-Vitvi17g00328\_t001 |  | \| \| |  |  |  |  |  |  |  |  |  |  |
| 1 | Vvi-Vitvi17g01407\_t001 |  | \| \| |  |  |  |  |  |  |  |  |  |  |
| 1 | Vvi-Vitvi17g01408\_t001 |  | \| \| |  |  |  |  |  |  |  |  |  |  |
| 1 | Vvi-Vitvi17g00329\_t001 |  | \| \| |  |  |  |  |  |  |  |  |  |  |
| 1 | Vvi-Vitvi17g04087\_t001 |  | \| \| |  |  |  |  |  |  |  |  |  |  |
| 1 | Vvi-Vitvi17g01409\_t001 |  | \| \| |  |  |  |  |  |  |  |  |  |  |
| 1 | Vvi-Vitvi17g00331\_t001 |  | Atr-ERN03938 |  |  |  |  |  |  |  |  |  |  |
| 0 | Vvi-Vitvi17g01410\_t001 |  |  |  |  |  |  |  |  |  |  |  |  |
| 0 | Vvi-Vitvi17g00332\_t001 |  |  |  |  |  |  |  |  |  |  |  |  |
| 0 | Vvi-Vitvi17g01411\_t001 |  |  |  |  |  |  |  |  |  |  |  |  |
| 0 | Vvi-Vitvi17g00333\_t001 |  |  |  |  |  |  |  |  |  |  |  |  |
| 0 | Vvi-Vitvi17g00334\_t001 |  |  |  |  |  |  |  |  |  |  |  |  |
| 0 | Vvi-Vitvi17g00335\_t001 |  |  |  |  |  |  |  |  |  |  |  |  |
| 0 | Vvi-Vitvi17g00336\_t001 |  |  |  |  |  |  |  |  |  |  |  |  |
| 0 | Vvi-Vitvi17g04088\_t001 |  |  |  |  |  |  |  |  |  |  |  |  |
| 0 | Vvi-Vitvi17g04089\_t001 |  |  |  |  |  |  |  |  |  |  |  |  |
| 0 | Vvi-Vitvi17g01414\_t001 |  |  |  |  |  |  |  |  |  |  |  |  |
| 0 | Vvi-Vitvi17g04090\_t001 |  |  |  |  |  |  |  |  |  |  |  |  |
| 0 | Vvi-Vitvi17g00339\_t001 |  |  |  |  |  |  |  |  |  |  |  |  |
| 0 | Vvi-Vitvi17g00340\_t001 |  |  |  |  |  |  |  |  |  |  |  |  |
| 0 | Vvi-Vitvi17g04091\_t001 |  |  |  |  |  |  |  |  |  |  |  |  |
| 0 | Vvi-Vitvi17g00343\_t001 |  |  |  |  |  |  |  |  |  |  |  |  |
| 0 | Vvi-Vitvi17g00344\_t001 |  |  |  |  |  |  |  |  |  |  |  |  |
| 0 | Vvi-Vitvi17g00347\_t001 |  |  |  |  |  |  |  |  |  |  |  |  |
| 0 | Vvi-Vitvi17g00348\_t001 |  |  |  |  |  |  |  |  |  |  |  |  |
| 0 | Vvi-Vitvi17g00349\_t001 |  |  |  |  |  |  |  |  |  |  |  |  |
| 0 | Vvi-Vitvi17g00350\_t001 |  |  |  |  |  |  |  |  |  |  |  |  |
| 0 | Vvi-Vitvi17g00351\_t002 |  |  |  |  |  |  |  |  |  |  |  |  |
| 0 | Vvi-Vitvi17g00355\_t001 |  |  |  |  |  |  |  |  |  |  |  |  |
| 0 | Vvi-Vitvi17g00356\_t001 |  |  |  |  |  |  |  |  |  |  |  |  |
| 0 | Vvi-Vitvi17g00359\_t001 |  |  |  |  |  |  |  |  |  |  |  |  |
| 0 | Vvi-Vitvi17g00360\_t001 |  |  |  |  |  |  |  |  |  |  |  |  |
| 0 | Vvi-Vitvi17g04092\_t001 |  |  |  |  |  |  |  |  |  |  |  |  |
| 0 | Vvi-Vitvi17g00362\_t001 |  |  |  |  |  |  |  |  |  |  |  |  |
| 0 | Vvi-Vitvi17g00363\_t001 |  |  |  |  |  |  |  |  |  |  |  |  |
| 0 | Vvi-Vitvi17g04093\_t002 |  |  |  |  |  |  |  |  |  |  |  |  |
| 0 | Vvi-Vitvi17g00365\_t002 |  |  |  |  |  |  |  |  |  |  |  |  |
| 0 | Vvi-Vitvi17g00366\_t001 |  |  |  |  |  |  |  |  |  |  |  |  |
| 0 | Vvi-Vitvi17g00367\_t001 |  |  |  |  |  |  |  |  |  |  |  |  |
| 0 | Vvi-Vitvi17g04094\_t001 |  |  |  |  |  |  |  |  |  |  |  |  |
| 0 | Vvi-Vitvi17g00368\_t001 |  |  |  |  |  |  |  |  |  |  |  |  |
| 0 | Vvi-Vitvi17g00371\_t001 |  |  |  |  |  |  |  |  |  |  |  |  |
| 0 | Vvi-Vitvi17g00372\_t001 |  |  |  |  |  |  |  |  |  |  |  |  |
| 0 | Vvi-Vitvi17g00373\_t001 |  |  |  |  |  |  |  |  |  |  |  |  |
| 0 | Vvi-Vitvi17g00374\_t001 |  |  |  |  |  |  |  |  |  |  |  |  |
| 0 | Vvi-Vitvi17g04095\_t001 |  |  |  |  |  |  |  |  |  |  |  |  |
| 0 | Vvi-Vitvi17g00376\_t001 |  |  |  |  |  |  |  |  |  |  |  |  |
| 0 | Vvi-Vitvi17g04096\_t001 |  |  |  |  |  |  |  |  |  |  |  |  |
| 0 | Vvi-Vitvi17g01420\_t001 |  |  |  |  |  |  |  |  |  |  |  |  |
| 0 | Vvi-Vitvi17g00379\_t001 |  |  |  |  |  |  |  |  |  |  |  |  |
| 0 | Vvi-Vitvi17g00380\_t001 |  |  |  |  |  |  |  |  |  |  |  |  |
| 0 | Vvi-Vitvi17g00381\_t002 |  |  |  |  |  |  |  |  |  |  |  |  |
| 0 | Vvi-Vitvi17g00382\_t003 |  |  |  |  |  |  |  |  |  |  |  |  |
| 0 | Vvi-Vitvi17g00383\_t001 |  |  |  |  |  |  |  |  |  |  |  |  |
| 0 | Vvi-Vitvi17g00384\_t001 |  |  |  |  |  |  |  |  |  |  |  |  |
| 0 | Vvi-Vitvi17g00385\_t001 |  |  |  |  |  |  |  |  |  |  |  |  |
| 0 | Vvi-Vitvi17g00386\_t001 |  |  |  |  |  |  |  |  |  |  |  |  |
| 0 | Vvi-Vitvi17g04097\_t001 |  |  |  |  |  |  |  |  |  |  |  |  |
| 0 | Vvi-Vitvi17g00388\_t001 |  |  |  |  |  |  |  |  |  |  |  |  |
| 0 | Vvi-Vitvi17g04098\_t001 |  |  |  |  |  |  |  |  |  |  |  |  |
| 0 | Vvi-Vitvi17g00390\_t001 |  |  |  |  |  |  |  |  |  |  |  |  |
| 0 | Vvi-Vitvi17g00391\_t001 |  |  |  |  |  |  |  |  |  |  |  |  |
| 0 | Vvi-Vitvi17g01421\_t001 |  |  |  |  |  |  |  |  |  |  |  |  |
| 0 | Vvi-Vitvi17g04099\_t001 |  |  |  |  |  |  |  |  |  |  |  |  |
| 0 | Vvi-Vitvi17g00392\_t001 |  |  |  |  |  |  |  |  |  |  |  |  |
| 0 | Vvi-Vitvi17g01422\_t001 |  |  |  |  |  |  |  |  |  |  |  |  |
| 0 | Vvi-Vitvi17g01423\_t001 |  |  |  |  |  |  |  |  |  |  |  |  |
| 0 | Vvi-Vitvi17g04100\_t001 |  |  |  |  |  |  |  |  |  |  |  |  |
| 0 | Vvi-Vitvi17g04101\_t001 |  |  |  |  |  |  |  |  |  |  |  |  |
| 0 | Vvi-Vitvi17g04102\_t001 |  |  |  |  |  |  |  |  |  |  |  |  |
| 0 | Vvi-Vitvi17g04103\_t001 |  |  |  |  |  |  |  |  |  |  |  |  |
| 0 | Vvi-Vitvi17g04104\_t001 |  |  |  |  |  |  |  |  |  |  |  |  |
| 0 | Vvi-Vitvi17g00395\_t001 |  |  |  |  |  |  |  |  |  |  |  |  |
| 0 | Vvi-Vitvi17g04105\_t001 |  |  |  |  |  |  |  |  |  |  |  |  |
| 0 | Vvi-Vitvi17g04106\_t001 |  |  |  |  |  |  |  |  |  |  |  |  |
| 0 | Vvi-Vitvi17g00397\_t001 |  |  |  |  |  |  |  |  |  |  |  |  |
| 0 | Vvi-Vitvi17g00398\_t001 |  |  |  |  |  |  |  |  |  |  |  |  |
| 0 | Vvi-Vitvi17g00399\_t001 |  |  |  |  |  |  |  |  |  |  |  |  |
| 0 | Vvi-Vitvi17g00400\_t001 |  |  |  |  |  |  |  |  |  |  |  |  |
| 0 | Vvi-Vitvi17g00402\_t001 |  |  |  |  |  |  |  |  |  |  |  |  |
| 0 | Vvi-Vitvi17g00403\_t001 |  |  |  |  |  |  |  |  |  |  |  |  |
| 0 | Vvi-Vitvi17g00405\_t001 |  |  |  |  |  |  |  |  |  |  |  |  |
| 0 | Vvi-Vitvi17g01425\_t001 |  |  |  |  |  |  |  |  |  |  |  |  |
| 0 | Vvi-Vitvi17g04107\_t001 |  |  |  |  |  |  |  |  |  |  |  |  |
| 1 | Vvi-Vitvi17g00406\_t001 |  | Atr-ERN05896 |  |  |  |  |  |  |  |  |  |  |
| 1 | Vvi-Vitvi17g04108\_t001 |  | \| \| |  |  |  |  |  |  |  |  |  |  |
| 1 | Vvi-Vitvi17g00407\_t001 |  | \| \| |  |  |  |  |  |  |  |  |  |  |
| 1 | Vvi-Vitvi17g00409\_t001 |  | Atr-ERN05891 |  |  |  |  |  |  |  |  |  |  |
| 1 | Vvi-Vitvi17g00411\_t001 |  | Atr-ERN05881 |  |  |  |  |  |  |  |  |  |  |
| 1 | Vvi-Vitvi17g00412\_t001 |  | \| \| |  |  |  |  |  |  |  |  |  |  |
| 1 | Vvi-Vitvi17g00413\_t001 |  | Atr-ERN05866 |  |  |  |  |  |  |  |  |  |  |
| 1 | Vvi-Vitvi17g00414\_t001 |  | Atr-ERN05855 |  |  |  |  |  |  |  |  |  |  |
| 1 | Vvi-Vitvi17g00415\_t001 |  | \| \| |  |  |  |  |  |  |  |  |  |  |
| 1 | Vvi-Vitvi17g01427\_t001 |  | \| \| |  |  |  |  |  |  |  |  |  |  |
| 1 | Vvi-Vitvi17g00416\_t001 |  | \| \| |  |  |  |  |  |  |  |  |  |  |
| 1 | Vvi-Vitvi17g00417\_t001 |  | \| \| |  |  |  |  |  |  |  |  |  |  |
| 1 | Vvi-Vitvi17g00418\_t001 |  | \| \| |  |  |  |  |  |  |  |  |  |  |
| 1 | Vvi-Vitvi17g00419\_t001 |  | \| \| |  |  |  |  |  |  |  |  |  |  |
| 1 | Vvi-Vitvi17g00420\_t001 |  | \| \| |  |  |  |  |  |  |  |  |  |  |
| 1 | Vvi-Vitvi17g00421\_t001 |  | \| \| |  |  |  |  |  |  |  |  |  |  |
| 1 | Vvi-Vitvi17g00422\_t001 |  | \| \| |  |  |  |  |  |  |  |  |  |  |
| 1 | Vvi-Vitvi17g00423\_t001 |  | \| \| |  |  |  |  |  |  |  |  |  |  |
| 1 | Vvi-Vitvi17g00424\_t001 |  | \| \| |  |  |  |  |  |  |  |  |  |  |
| 1 | Vvi-Vitvi17g00425\_t001 |  | \| \| |  |  |  |  |  |  |  |  |  |  |
| 1 | Vvi-Vitvi17g00426\_t001 |  | Atr-ERN05837 |  |  |  |  |  |  |  |  |  |  |
| 1 | Vvi-Vitvi17g00427\_t001 |  | \| \| |  |  |  |  |  |  |  |  |  |  |
| 1 | Vvi-Vitvi17g00428\_t001 |  | \| \| |  |  |  |  |  |  |  |  |  |  |
| 1 | Vvi-Vitvi17g04109\_t001 |  | \| \| |  |  |  |  |  |  |  |  |  |  |
| 1 | Vvi-Vitvi17g04110\_t001 |  | \| \| |  |  |  |  |  |  |  |  |  |  |
| 1 | Vvi-Vitvi17g01428\_t001 |  | \| \| |  |  |  |  |  |  |  |  |  |  |
| 1 | Vvi-Vitvi17g04111\_t001 |  | \| \| |  |  |  |  |  |  |  |  |  |  |
| 1 | Vvi-Vitvi17g04112\_t001 |  | \| \| |  |  |  |  |  |  |  |  |  |  |
| 1 | Vvi-Vitvi17g01429\_t001 |  | \| \| |  |  |  |  |  |  |  |  |  |  |
| 1 | Vvi-Vitvi17g00430\_t001 |  | Atr-ERN05825 |  |  |  |  |  |  |  |  |  |  |
| 1 | Vvi-Vitvi17g04113\_t001 |  | \| \| |  |  |  |  |  |  |  |  |  |  |
| 1 | Vvi-Vitvi17g00431\_t001 |  | \| \| |  |  |  |  |  |  |  |  |  |  |
| 1 | Vvi-Vitvi17g04114\_t001 |  | \| \| |  |  |  |  |  |  |  |  |  |  |
| 1 | Vvi-Vitvi17g04115\_t001 |  | \| \| |  |  |  |  |  |  |  |  |  |  |
| 1 | Vvi-Vitvi17g00432\_t001 |  | \| \| |  |  |  |  |  |  |  |  |  |  |
| 1 | Vvi-Vitvi17g04116\_t001 |  | \| \| |  |  |  |  |  |  |  |  |  |  |
| 1 | Vvi-Vitvi17g04117\_t001 |  | \| \| |  |  |  |  |  |  |  |  |  |  |
| 1 | Vvi-Vitvi17g04118\_t001 |  | \| \| |  |  |  |  |  |  |  |  |  |  |
| 1 | Vvi-Vitvi17g04119\_t001 |  | \| \| |  |  |  |  |  |  |  |  |  |  |
| 1 | Vvi-Vitvi17g00433\_t001 |  | \| \| |  |  |  |  |  |  |  |  |  |  |
| 1 | Vvi-Vitvi17g04120\_t001 |  | Atr-ERN05817 |  |  |  |  |  |  |  |  |  |  |
| 1 | Vvi-Vitvi17g00434\_t001 |  | \| \| |  |  |  |  |  |  |  |  |  |  |
| 1 | Vvi-Vitvi17g00435\_t001 |  | Atr-ERN05807 |  |  |  |  |  |  |  |  |  |  |
| 1 | Vvi-Vitvi17g00436\_t001 |  | Atr-ERN05801 |  |  |  |  |  |  |  |  |  |  |
| 0 | Vvi-Vitvi17g04121\_t001 |  |  |  |  |  |  |  |  |  |  |  |  |
| 0 | Vvi-Vitvi17g04122\_t001 |  |  |  |  |  |  |  |  |  |  |  |  |
| 0 | Vvi-Vitvi17g04123\_t001 |  |  |  |  |  |  |  |  |  |  |  |  |
| 0 | Vvi-Vitvi17g00438\_t001 |  |  |  |  |  |  |  |  |  |  |  |  |
| 0 | Vvi-Vitvi17g00439\_t001 |  |  |  |  |  |  |  |  |  |  |  |  |
| 1 | Vvi-Vitvi17g00440\_t001 |  | Atr-ERN05762 |  |  |  |  |  |  |  |  |  |  |
| 1 | Vvi-Vitvi17g00441\_t001 |  | Atr-ERN05755 |  |  |  |  |  |  |  |  |  |  |
| 1 | Vvi-Vitvi17g04124\_t001 |  | \| \| |  |  |  |  |  |  |  |  |  |  |
| 1 | Vvi-Vitvi17g00442\_t001 |  | Atr-ERN05752 |  |  |  |  |  |  |  |  |  |  |
| 1 | Vvi-Vitvi17g00443\_t001 |  | Atr-ERN05750 |  |  |  |  |  |  |  |  |  |  |
| 1 | Vvi-Vitvi17g00444\_t004 |  | Atr-ERN05746 |  |  |  |  |  |  |  |  |  |  |
| 1 | Vvi-Vitvi17g00445\_t001 |  | \| \| |  |  |  |  |  |  |  |  |  |  |
| 1 | Vvi-Vitvi17g04125\_t001 |  | \| \| |  |  |  |  |  |  |  |  |  |  |
| 1 | Vvi-Vitvi17g01433\_t002 |  | \| \| |  |  |  |  |  |  |  |  |  |  |
| 1 | Vvi-Vitvi17g00447\_t001 |  | Atr-ERN05743 |  |  |  |  |  |  |  |  |  |  |
| 1 | Vvi-Vitvi17g04126\_t001 |  | \| \| |  |  |  |  |  |  |  |  |  |  |
| 1 | Vvi-Vitvi17g01434\_t001 |  | Atr-ERN05737 |  |  |  |  |  |  |  |  |  |  |
| 1 | Vvi-Vitvi17g04127\_t001 |  | \| \| |  |  |  |  |  |  |  |  |  |  |
| 1 | Vvi-Vitvi17g01435\_t001 |  | Atr-ERN05735 |  |  |  |  |  |  |  |  |  |  |
| 1 | Vvi-Vitvi17g01436\_t001 |  | \| \| |  |  |  |  |  |  |  |  |  |  |
| 1 | Vvi-Vitvi17g00450\_t001 |  | \| \| |  |  |  |  |  |  |  |  |  |  |
| 1 | Vvi-Vitvi17g00451\_t001 |  | Atr-ERN05734 |  |  |  |  |  |  |  |  |  |  |
| 1 | Vvi-Vitvi17g00452\_t001 |  | Atr-ERN17773 |  |  |  |  |  |  |  |  |  |  |
| 1 | Vvi-Vitvi17g00453\_t001 |  | \| \| |  |  |  |  |  |  |  |  |  |  |
| 1 | Vvi-Vitvi17g01437\_t001 |  | \| \| |  |  |  |  |  |  |  |  |  |  |
| 1 | Vvi-Vitvi17g00454\_t001 |  | \| \| |  |  |  |  |  |  |  |  |  |  |
| 1 | Vvi-Vitvi17g00455\_t001 |  | \| \| |  |  |  |  |  |  |  |  |  |  |
| 1 | Vvi-Vitvi17g00457\_t001 |  | Atr-ERN17781 |  |  |  |  |  |  |  |  |  |  |
| 1 | Vvi-Vitvi17g04128\_t001 |  | \| \| |  |  |  |  |  |  |  |  |  |  |
| 1 | Vvi-Vitvi17g04129\_t001 |  | \| \| |  |  |  |  |  |  |  |  |  |  |
| 1 | Vvi-Vitvi17g00460\_t001 |  | \| \| |  |  |  |  |  |  |  |  |  |  |
| 1 | Vvi-Vitvi17g00461\_t001 |  | \| \| |  |  |  |  |  |  |  |  |  |  |
| 1 | Vvi-Vitvi17g04130\_t001 |  | Atr-ERN17793 |  |  |  |  |  |  |  |  |  |  |
| 1 | Vvi-Vitvi17g00464\_t001 |  | \| \| |  |  |  |  |  |  |  |  |  |  |
| 1 | Vvi-Vitvi17g04131\_t001 |  | \| \| |  |  |  |  |  |  |  |  |  |  |
| 1 | Vvi-Vitvi17g00466\_t001 |  | \| \| |  |  |  |  |  |  |  |  |  |  |
| 1 | Vvi-Vitvi17g00468\_t001 |  | \| \| |  |  |  |  |  |  |  |  |  |  |
| 1 | Vvi-Vitvi17g00469\_t001 |  | Atr-ERN17818 |  |  |  |  |  |  |  |  |  |  |
| 1 | Vvi-Vitvi17g00470\_t001 |  | Atr-ERN17823 |  |  |  |  |  |  |  |  |  |  |
| 1 | Vvi-Vitvi17g00471\_t001 |  | \| \| |  |  |  |  |  |  |  |  |  |  |
| 1 | Vvi-Vitvi17g01438\_t001 |  | \| \| |  |  |  |  |  |  |  |  |  |  |
| 1 | Vvi-Vitvi17g01439\_t001 |  | \| \| |  |  |  |  |  |  |  |  |  |  |
| 1 | Vvi-Vitvi17g04132\_t001 |  | \| \| |  |  |  |  |  |  |  |  |  |  |
| 1 | Vvi-Vitvi17g00473\_t001 |  | Atr-ERN17843 |  |  |  |  |  |  |  |  |  |  |
| 1 | Vvi-Vitvi17g01440\_t001 |  | Atr-ERN17867 |  |  |  |  |  |  |  |  |  |  |
| 1 | Vvi-Vitvi17g04133\_t001 |  | Atr-ERN17880 |  |  |  |  |  |  |  |  |  |  |
| 1 | Vvi-Vitvi17g04134\_t001 |  | \| \| |  |  |  |  |  |  |  |  |  |  |
| 1 | Vvi-Vitvi17g00478\_t001 |  | \| \| |  |  |  |  |  |  |  |  |  |  |
| 1 | Vvi-Vitvi17g00479\_t001 |  | \| \| |  |  |  |  |  |  |  |  |  |  |
| 1 | Vvi-Vitvi17g00480\_t001 |  | \| \| |  |  |  |  |  |  |  |  |  |  |
| 1 | Vvi-Vitvi17g00482\_t001 |  | Atr-ERN17889 |  |  |  |  |  |  |  |  |  |  |
| 1 | Vvi-Vitvi17g00483\_t001 |  | \| \| |  |  |  |  |  |  |  |  |  |  |
| 1 | Vvi-Vitvi17g04135\_t001 |  | \| \| |  |  |  |  |  |  |  |  |  |  |
| 1 | Vvi-Vitvi17g00484\_t001 |  | Atr-ERN17891 |  |  |  |  |  |  |  |  |  |  |
| 1 | Vvi-Vitvi17g00485\_t001 |  | Atr-ERN17906 |  |  |  |  |  |  |  |  |  |  |
| 1 | Vvi-Vitvi17g00486\_t001 |  | \| \| |  |  |  |  |  |  |  |  |  |  |
| 1 | Vvi-Vitvi17g00487\_t001 |  | Atr-ERN17908 |  |  |  |  |  |  |  |  |  |  |
| 1 | Vvi-Vitvi17g00488\_t001 |  | Atr-ERN17915 |  |  |  |  |  |  |  |  |  |  |
| 1 | Vvi-Vitvi17g00489\_t001 |  | Atr-ERN17916 |  |  |  |  |  |  |  |  |  |  |
| 1 | Vvi-Vitvi17g00490\_t001 |  | Atr-ERN07562 |  |  |  |  |  |  |  |  |  |  |
| 1 | Vvi-Vitvi17g04136\_t001 |  | \| \| |  |  |  |  |  |  |  |  |  |  |
| 1 | Vvi-Vitvi17g00493\_t003 |  | Atr-ERN07560 |  |  |  |  |  |  |  |  |  |  |
| 1 | Vvi-Vitvi17g00494\_t001 |  | Atr-ERN07559 |  |  |  |  |  |  |  |  |  |  |
| 1 | Vvi-Vitvi17g00495\_t001 |  | Atr-ERN07554 |  |  |  |  |  |  |  |  |  |  |
| 1 | Vvi-Vitvi17g01446\_t001 |  | \| \| |  |  |  |  |  |  |  |  |  |  |
| 1 | Vvi-Vitvi17g00496\_t002 |  | Atr-ERN07552 |  |  |  |  |  |  |  |  |  |  |
| 1 | Vvi-Vitvi17g00497\_t001 |  | Atr-ERN07551 |  |  |  |  |  |  |  |  |  |  |
| 1 | Vvi-Vitvi17g00498\_t001 |  | \| \| |  |  |  |  |  |  |  |  |  |  |
| 1 | Vvi-Vitvi17g01447\_t001 |  | \| \| |  |  |  |  |  |  |  |  |  |  |
| 1 | Vvi-Vitvi17g04137\_t001 |  | \| \| |  |  |  |  |  |  |  |  |  |  |
| 1 | Vvi-Vitvi17g01448\_t001 |  | Atr-ERN07542 |  |  |  |  |  |  |  |  |  |  |
| 0 | Vvi-Vitvi17g04138\_t001 |  |  |  |  |  |  |  |  |  |  |  |  |
| 0 | Vvi-Vitvi17g00499\_t001 |  |  |  |  |  |  |  |  |  |  |  |  |
| 0 | Vvi-Vitvi17g04139\_t001 |  |  |  |  |  |  |  |  |  |  |  |  |
| 0 | Vvi-Vitvi17g04140\_t001 |  |  |  |  |  |  |  |  |  |  |  |  |
| 0 | Vvi-Vitvi17g01449\_t001 |  |  |  |  |  |  |  |  |  |  |  |  |
| 0 | Vvi-Vitvi17g00503\_t001 |  |  |  |  |  |  |  |  |  |  |  |  |
| 0 | Vvi-Vitvi17g00504\_t001 |  |  |  |  |  |  |  |  |  |  |  |  |
| 0 | Vvi-Vitvi17g00505\_t001 |  |  |  |  |  |  |  |  |  |  |  |  |
| 0 | Vvi-Vitvi17g00506\_t001 |  |  |  |  |  |  |  |  |  |  |  |  |
| 0 | Vvi-Vitvi17g01451\_t001 |  |  |  |  |  |  |  |  |  |  |  |  |
| 0 | Vvi-Vitvi17g00507\_t001 |  |  |  |  |  |  |  |  |  |  |  |  |
| 0 | Vvi-Vitvi17g00508\_t001 |  |  |  |  |  |  |  |  |  |  |  |  |
| 0 | Vvi-Vitvi17g00509\_t001 |  |  |  |  |  |  |  |  |  |  |  |  |
| 0 | Vvi-Vitvi17g00510\_t001 |  |  |  |  |  |  |  |  |  |  |  |  |
| 0 | Vvi-Vitvi17g00511\_t001 |  |  |  |  |  |  |  |  |  |  |  |  |
| 0 | Vvi-Vitvi17g04141\_t001 |  |  |  |  |  |  |  |  |  |  |  |  |
| 0 | Vvi-Vitvi17g01452\_t001 |  |  |  |  |  |  |  |  |  |  |  |  |
| 0 | Vvi-Vitvi17g00512\_t001 |  |  |  |  |  |  |  |  |  |  |  |  |
| 0 | Vvi-Vitvi17g00514\_t001 |  |  |  |  |  |  |  |  |  |  |  |  |
| 0 | Vvi-Vitvi17g00515\_t001 |  |  |  |  |  |  |  |  |  |  |  |  |
| 0 | Vvi-Vitvi17g00516\_t001 |  |  |  |  |  |  |  |  |  |  |  |  |
| 0 | Vvi-Vitvi17g00518\_t001 |  |  |  |  |  |  |  |  |  |  |  |  |
| 0 | Vvi-Vitvi17g00519\_t001 |  |  |  |  |  |  |  |  |  |  |  |  |
| 0 | Vvi-Vitvi17g00520\_t001 |  |  |  |  |  |  |  |  |  |  |  |  |
| 0 | Vvi-Vitvi17g00521\_t001 |  |  |  |  |  |  |  |  |  |  |  |  |
| 0 | Vvi-Vitvi17g04142\_t001 |  |  |  |  |  |  |  |  |  |  |  |  |
| 0 | Vvi-Vitvi17g00522\_t001 |  |  |  |  |  |  |  |  |  |  |  |  |
| 0 | Vvi-Vitvi17g04143\_t001 |  |  |  |  |  |  |  |  |  |  |  |  |
| 0 | Vvi-Vitvi17g00523\_t001 |  |  |  |  |  |  |  |  |  |  |  |  |
| 0 | Vvi-Vitvi17g00524\_t002 |  |  |  |  |  |  |  |  |  |  |  |  |
| 0 | Vvi-Vitvi17g00527\_t001 |  |  |  |  |  |  |  |  |  |  |  |  |
| 0 | Vvi-Vitvi17g00528\_t001 |  |  |  |  |  |  |  |  |  |  |  |  |
| 0 | Vvi-Vitvi17g04144\_t001 |  |  |  |  |  |  |  |  |  |  |  |  |
| 0 | Vvi-Vitvi17g00530\_t001 |  |  |  |  |  |  |  |  |  |  |  |  |
| 0 | Vvi-Vitvi17g00532\_t001 |  |  |  |  |  |  |  |  |  |  |  |  |
| 0 | Vvi-Vitvi17g04145\_t001 |  |  |  |  |  |  |  |  |  |  |  |  |
| 0 | Vvi-Vitvi17g00533\_t001 |  |  |  |  |  |  |  |  |  |  |  |  |
| 0 | Vvi-Vitvi17g00534\_t001 |  |  |  |  |  |  |  |  |  |  |  |  |
| 0 | Vvi-Vitvi17g00535\_t001 |  |  |  |  |  |  |  |  |  |  |  |  |
| 0 | Vvi-Vitvi17g04146\_t001 |  |  |  |  |  |  |  |  |  |  |  |  |
| 0 | Vvi-Vitvi17g04147\_t001 |  |  |  |  |  |  |  |  |  |  |  |  |
| 0 | Vvi-Vitvi17g04148\_t001 |  |  |  |  |  |  |  |  |  |  |  |  |
| 0 | Vvi-Vitvi17g00537\_t001 |  |  |  |  |  |  |  |  |  |  |  |  |
| 0 | Vvi-Vitvi17g00538\_t001 |  |  |  |  |  |  |  |  |  |  |  |  |
| 0 | Vvi-Vitvi17g00539\_t001 |  |  |  |  |  |  |  |  |  |  |  |  |
| 0 | Vvi-Vitvi17g04149\_t001 |  |  |  |  |  |  |  |  |  |  |  |  |
| 0 | Vvi-Vitvi17g00540\_t001 |  |  |  |  |  |  |  |  |  |  |  |  |
| 1 | Vvi-Vitvi17g00541\_t001 |  | Atr-ERN03774 |  |  |  |  |  |  |  |  |  |  |
| 1 | Vvi-Vitvi17g00543\_t001 |  | Atr-ERN03776 |  |  |  |  |  |  |  |  |  |  |
| 1 | Vvi-Vitvi17g00544\_t001 |  | \| \| |  |  |  |  |  |  |  |  |  |  |
| 1 | Vvi-Vitvi17g00545\_t001 |  | \| \| |  |  |  |  |  |  |  |  |  |  |
| 1 | Vvi-Vitvi17g00546\_t002 |  | Atr-ERN03788 |  |  |  |  |  |  |  |  |  |  |
| 1 | Vvi-Vitvi17g00547\_t001 |  | Atr-ERN03790 |  |  |  |  |  |  |  |  |  |  |
| 1 | Vvi-Vitvi17g04150\_t001 |  | \| \| |  |  |  |  |  |  |  |  |  |  |
| 1 | Vvi-Vitvi17g00548\_t001 |  | Atr-ERN03792 |  |  |  |  |  |  |  |  |  |  |
| 1 | Vvi-Vitvi17g01454\_t001 |  | \| \| |  |  |  |  |  |  |  |  |  |  |
| 1 | Vvi-Vitvi17g01455\_t001 |  | \| \| |  |  |  |  |  |  |  |  |  |  |
| 1 | Vvi-Vitvi17g01457\_t001 |  | \| \| |  |  |  |  |  |  |  |  |  |  |
| 1 | Vvi-Vitvi17g04151\_t001 |  | \| \| |  |  |  |  |  |  |  |  |  |  |
| 1 | Vvi-Vitvi17g00550\_t001 |  | Atr-ERN03796 |  |  |  |  |  |  |  |  |  |  |
| 1 | Vvi-Vitvi17g00551\_t002 |  | Atr-ERN03797 |  |  |  |  |  |  |  |  |  |  |
| 1 | Vvi-Vitvi17g01459\_t001 |  | Atr-ERN03800 |  |  |  |  |  |  |  |  |  |  |
| 1 | Vvi-Vitvi17g04152\_t001 |  | \| \| |  |  |  |  |  |  |  |  |  |  |
| 1 | Vvi-Vitvi17g00552\_t001 |  | Atr-ERN03803 |  |  |  |  |  |  |  |  |  |  |
| 1 | Vvi-Vitvi17g00553\_t001 |  | Atr-ERN03809 |  |  |  |  |  |  |  |  |  |  |
| 1 | Vvi-Vitvi17g00554\_t001 |  | Atr-ERN03813 |  |  |  |  |  |  |  |  |  |  |
| 1 | Vvi-Vitvi17g00555\_t001 |  | Atr-ERN03815 |  |  |  |  |  |  |  |  |  |  |
| 1 | Vvi-Vitvi17g00556\_t001 |  | Atr-ERN03816 |  |  |  |  |  |  |  |  |  |  |
| 1 | Vvi-Vitvi17g04153\_t001 |  | Atr-ERN03822 |  |  |  |  |  |  |  |  |  |  |
| 1 | Vvi-Vitvi17g00558\_t001 |  | Atr-ERN03825 |  |  |  |  |  |  |  |  |  |  |
| 1 | Vvi-Vitvi17g04154\_t001 |  | \| \| |  |  |  |  |  |  |  |  |  |  |
| 1 | Vvi-Vitvi17g00560\_t001 |  | Atr-ERN03828 |  |  |  |  |  |  |  |  |  |  |
| 1 | Vvi-Vitvi17g00561\_t001 |  | Atr-ERN03831 |  |  |  |  |  |  |  |  |  |  |
| 0 | Vvi-Vitvi17g00562\_t001 |  |  |  |  |  |  |  |  |  |  |  |  |
| 0 | Vvi-Vitvi17g00563\_t001 |  |  |  |  |  |  |  |  |  |  |  |  |
| 1 | Vvi-Vitvi17g00565\_t001 |  | Atr-ERM94378 |  |  |  |  |  |  |  |  |  |  |
| 1 | Vvi-Vitvi17g00566\_t001 |  | \| \| |  |  |  |  |  |  |  |  |  |  |
| 1 | Vvi-Vitvi17g00567\_t001 |  | Atr-ERM94383 |  |  |  |  |  |  |  |  |  |  |
| 1 | Vvi-Vitvi17g00568\_t001 |  | Atr-ERM94384 |  |  |  |  |  |  |  |  |  |  |
| 1 | Vvi-Vitvi17g00569\_t001 |  | Atr-ERM94404 |  |  |  |  |  |  |  |  |  |  |
| 1 | Vvi-Vitvi17g00570\_t001 |  | Atr-ERM94407 |  |  |  |  |  |  |  |  |  |  |
| 1 | Vvi-Vitvi17g00571\_t001 |  | Atr-ERM94408 |  |  |  |  |  |  |  |  |  |  |
| 1 | Vvi-Vitvi17g00573\_t001 |  | \| \| |  |  |  |  |  |  |  |  |  |  |
| 1 | Vvi-Vitvi17g00574\_t001 |  | \| \| |  |  |  |  |  |  |  |  |  |  |
| 1 | Vvi-Vitvi17g04155\_t001 |  | Atr-ERM94413 |  |  |  |  |  |  |  |  |  |  |
| 1 | Vvi-Vitvi17g04156\_t001 |  | Atr-ERM94419 |  |  |  |  |  |  |  |  |  |  |
| 1 | Vvi-Vitvi17g00577\_t001 |  | Atr-ERM94426 |  |  |  |  |  |  |  |  |  |  |
| 1 | Vvi-Vitvi17g00578\_t001 |  | Atr-ERM94428 |  |  |  |  |  |  |  |  |  |  |
| 1 | Vvi-Vitvi17g00579\_t001 |  | Atr-ERM94429 |  |  |  |  |  |  |  |  |  |  |
| 1 | Vvi-Vitvi17g00581\_t001 |  | Atr-ERM94431 |  |  |  |  |  |  |  |  |  |  |
| 1 | Vvi-Vitvi17g00582\_t001 |  | Atr-ERM94443 |  |  |  |  |  |  |  |  |  |  |
| 1 | Vvi-Vitvi17g00583\_t001 |  | Atr-ERM94445 |  |  |  |  |  |  |  |  |  |  |
| 1 | Vvi-Vitvi17g00584\_t001 |  | Atr-ERM94450 |  |  |  |  |  |  |  |  |  |  |
| 1 | Vvi-Vitvi17g00586\_t001 |  | Atr-ERM94457 |  |  |  |  |  |  |  |  |  |  |
| 1 | Vvi-Vitvi17g00587\_t001 |  | \| \| |  |  |  |  |  |  |  |  |  |  |
| 1 | Vvi-Vitvi17g04157\_t001 |  | \| \| |  |  |  |  |  |  |  |  |  |  |
| 1 | Vvi-Vitvi17g04158\_t001 |  | \| \| |  |  |  |  |  |  |  |  |  |  |
| 1 | Vvi-Vitvi17g00590\_t001 |  | \| \| |  |  |  |  |  |  |  |  |  |  |
| 1 | Vvi-Vitvi17g00591\_t001 |  | \| \| |  |  |  |  |  |  |  |  |  |  |
| 1 | Vvi-Vitvi17g01465\_t001 |  | Atr-ERM94463 |  |  |  |  |  |  |  |  |  |  |
| 1 | Vvi-Vitvi17g01466\_t001 |  | \| \| |  |  |  |  |  |  |  |  |  |  |
| 1 | Vvi-Vitvi17g01467\_t001 |  | \| \| |  |  |  |  |  |  |  |  |  |  |
| 1 | Vvi-Vitvi17g04159\_t001 |  | \| \| |  |  |  |  |  |  |  |  |  |  |
| 1 | Vvi-Vitvi17g00592\_t001 |  | Atr-ERM94464 |  |  |  |  |  |  |  |  |  |  |
| 1 | Vvi-Vitvi17g04160\_t001 |  | \| \| |  |  |  |  |  |  |  |  |  |  |
| 1 | Vvi-Vitvi17g04161\_t001 |  | \| \| |  |  |  |  |  |  |  |  |  |  |
| 1 | Vvi-Vitvi17g00593\_t001 |  | \| \| |  |  |  |  |  |  |  |  |  |  |
| 1 | Vvi-Vitvi17g04162\_t001 |  | \| \| |  |  |  |  |  |  |  |  |  |  |
| 1 | Vvi-Vitvi17g00596\_t001 |  | \| \| |  |  |  |  |  |  |  |  |  |  |
| 1 | Vvi-Vitvi17g00597\_t001 |  | Atr-ERM94471 |  |  |  |  |  |  |  |  |  |  |
| 1 | Vvi-Vitvi17g00598\_t001 |  | Atr-ERM94481 |  |  |  |  |  |  |  |  |  |  |
| 1 | Vvi-Vitvi17g00600\_t001 |  | \| \| |  |  |  |  |  |  |  |  |  |  |
| 1 | Vvi-Vitvi17g00601\_t001 |  | \| \| |  |  |  |  |  |  |  |  |  |  |
| 1 | Vvi-Vitvi17g04163\_t001 |  | \| \| |  |  |  |  |  |  |  |  |  |  |
| 1 | Vvi-Vitvi17g00602\_t001 |  | \| \| |  |  |  |  |  |  |  |  |  |  |
| 1 | Vvi-Vitvi17g04164\_t001 |  | \| \| |  |  |  |  |  |  |  |  |  |  |
| 1 | Vvi-Vitvi17g00603\_t001 |  | \| \| |  |  |  |  |  |  |  |  |  |  |
| 1 | Vvi-Vitvi17g00604\_t001 |  | \| \| |  |  |  |  |  |  |  |  |  |  |
| 1 | Vvi-Vitvi17g00605\_t001 |  | \| \| |  |  |  |  |  |  |  |  |  |  |
| 1 | Vvi-Vitvi17g04165\_t001 |  | \| \| |  |  |  |  |  |  |  |  |  |  |
| 1 | Vvi-Vitvi17g00607\_t001 |  | Atr-ERM94496 |  |  |  |  |  |  |  |  |  |  |
| 1 | Vvi-Vitvi17g04166\_t001 |  | \| \| |  |  |  |  |  |  |  |  |  |  |
| 1 | Vvi-Vitvi17g00608\_t001 |  | Atr-ERM94498 |  |  |  |  |  |  |  |  |  |  |
| 1 | Vvi-Vitvi17g00609\_t001 |  | Atr-ERM94499 |  |  |  |  |  |  |  |  |  |  |
| 1 | Vvi-Vitvi17g01470\_t001 |  | \| \| |  |  |  |  |  |  |  |  |  |  |
| 1 | Vvi-Vitvi17g00611\_t001 |  | Atr-ERM94520 |  |  |  |  |  |  |  |  |  |  |
| 1 | Vvi-Vitvi17g04167\_t001 |  | \| \| |  |  |  |  |  |  |  |  |  |  |
| 1 | Vvi-Vitvi17g00612\_t001 |  | Atr-ERM94522 |  |  |  |  |  |  |  |  |  |  |
| 1 | Vvi-Vitvi17g04168\_t001 |  | \| \| |  |  |  |  |  |  |  |  |  |  |
| 1 | Vvi-Vitvi17g01471\_t001 |  | Atr-ERM94524 |  |  |  |  |  |  |  |  |  |  |
| 1 | Vvi-Vitvi17g04169\_t001 |  | \| \| |  |  |  |  |  |  |  |  |  |  |
| 1 | Vvi-Vitvi17g00613\_t001 |  | \| \| |  |  |  |  |  |  |  |  |  |  |
| 1 | Vvi-Vitvi17g04170\_t001 |  | \| \| |  |  |  |  |  |  |  |  |  |  |
| 1 | Vvi-Vitvi17g00614\_t001 |  | Atr-ERM94528 |  |  |  |  |  |  |  |  |  |  |
| 1 | Vvi-Vitvi17g00615\_t001 |  | Atr-ERM94529 |  |  |  |  |  |  |  |  |  |  |
| 1 | Vvi-Vitvi17g00616\_t001 |  | \| \| |  |  |  |  |  |  |  |  |  |  |
| 1 | Vvi-Vitvi17g00617\_t001 |  | Atr-ERM94538 |  |  |  |  |  |  |  |  |  |  |
| 1 | Vvi-Vitvi17g04171\_t001 |  | Atr-ERM94539 |  |  |  |  |  |  |  |  |  |  |
| 1 | Vvi-Vitvi17g04172\_t001 |  | \| \| |  |  |  |  |  |  |  |  |  |  |
| 1 | Vvi-Vitvi17g00621\_t001 |  | \| \| |  |  |  |  |  |  |  |  |  |  |
| 1 | Vvi-Vitvi17g01474\_t001 |  | \| \| |  |  |  |  |  |  |  |  |  |  |
| 1 | Vvi-Vitvi17g00622\_t001 |  | Atr-ERM94547 |  |  |  |  |  |  |  |  |  |  |
| 1 | Vvi-Vitvi17g00623\_t001 |  | Atr-ERM94557 |  |  |  |  |  |  |  |  |  |  |
| 1 | Vvi-Vitvi17g00624\_t001 |  | Atr-ERM94558 |  |  |  |  |  |  |  |  |  |  |
| 1 | Vvi-Vitvi17g04173\_t001 |  | Atr-ERM94560 |  |  |  |  |  |  |  |  |  |  |
| 1 | Vvi-Vitvi17g00627\_t001 |  | Atr-ERM94561 |  |  |  |  |  |  |  |  |  |  |
| 1 | Vvi-Vitvi17g00629\_t001 |  | \| \| |  |  |  |  |  |  |  |  |  |  |
| 1 | Vvi-Vitvi17g04174\_t001 |  | \| \| |  |  |  |  |  |  |  |  |  |  |
| 1 | Vvi-Vitvi17g00630\_t001 |  | Atr-ERM94565 |  |  |  |  |  |  |  |  |  |  |
| 1 | Vvi-Vitvi17g00631\_t001 |  | \| \| |  |  |  |  |  |  |  |  |  |  |
| 1 | Vvi-Vitvi17g00632\_t001 |  | \| \| |  |  |  |  |  |  |  |  |  |  |
| 1 | Vvi-Vitvi17g00633\_t001 |  | \| \| |  |  |  |  |  |  |  |  |  |  |
| 1 | Vvi-Vitvi17g00634\_t001 |  | \| \| |  |  |  |  |  |  |  |  |  |  |
| 1 | Vvi-Vitvi17g00635\_t001 |  | Atr-ERM94571 |  |  |  |  |  |  |  |  |  |  |
| 0 | Vvi-Vitvi17g00636\_t001 |  |  |  |  |  |  |  |  |  |  |  |  |
| 0 | Vvi-Vitvi17g00637\_t001 |  |  |  |  |  |  |  |  |  |  |  |  |
| 0 | Vvi-Vitvi17g00638\_t001 |  |  |  |  |  |  |  |  |  |  |  |  |
| 0 | Vvi-Vitvi17g04175\_t001 |  |  |  |  |  |  |  |  |  |  |  |  |
| 0 | Vvi-Vitvi17g00640\_t001 |  |  |  |  |  |  |  |  |  |  |  |  |
| 0 | Vvi-Vitvi17g04176\_t001 |  |  |  |  |  |  |  |  |  |  |  |  |
| 0 | Vvi-Vitvi17g00641\_t001 |  |  |  |  |  |  |  |  |  |  |  |  |
| 0 | Vvi-Vitvi17g00642\_t001 |  |  |  |  |  |  |  |  |  |  |  |  |
| 0 | Vvi-Vitvi17g01477\_t001 |  |  |  |  |  |  |  |  |  |  |  |  |
| 0 | Vvi-Vitvi17g04177\_t001 |  |  |  |  |  |  |  |  |  |  |  |  |
| 1 | Vvi-Vitvi17g00643\_t001 |  | Atr-ERN06498 |  |  |  |  |  |  |  |  |  |  |
| 1 | Vvi-Vitvi17g00644\_t001 |  | \| \| |  |  |  |  |  |  |  |  |  |  |
| 1 | Vvi-Vitvi17g00645\_t001 |  | Atr-ERN06509 |  |  |  |  |  |  |  |  |  |  |
| 1 | Vvi-Vitvi17g00646\_t001 |  | Atr-ERN06513 |  |  |  |  |  |  |  |  |  |  |
| 1 | Vvi-Vitvi17g00648\_t001 |  | Atr-ERN06520 |  |  |  |  |  |  |  |  |  |  |
| 1 | Vvi-Vitvi17g00649\_t001 |  | Atr-ERN06522 |  |  |  |  |  |  |  |  |  |  |
| 1 | Vvi-Vitvi17g00650\_t001 |  | Atr-ERN06527 |  |  |  |  |  |  |  |  |  |  |
| 1 | Vvi-Vitvi17g00651\_t001 |  | Atr-ERN06535 |  |  |  |  |  |  |  |  |  |  |
| 1 | Vvi-Vitvi17g04178\_t001 |  | \| \| |  |  |  |  |  |  |  |  |  |  |
| 1 | Vvi-Vitvi17g01479\_t001 |  | \| \| |  |  |  |  |  |  |  |  |  |  |
| 1 | Vvi-Vitvi17g04179\_t001 |  | \| \| |  |  |  |  |  |  |  |  |  |  |
| 1 | Vvi-Vitvi17g01481\_t001 |  | \| \| |  |  |  |  |  |  |  |  |  |  |
| 1 | Vvi-Vitvi17g04180\_t001 |  | \| \| |  |  |  |  |  |  |  |  |  |  |
| 1 | Vvi-Vitvi17g04181\_t001 |  | \| \| |  |  |  |  |  |  |  |  |  |  |
| 1 | Vvi-Vitvi17g04182\_t001 |  | \| \| |  |  |  |  |  |  |  |  |  |  |
| 1 | Vvi-Vitvi17g04183\_t001 |  | Atr-ERN06546 |  |  |  |  |  |  |  |  |  |  |
| 0 | Vvi-Vitvi17g04184\_t001 |  |  |  |  |  |  |  |  |  |  |  |  |
| 0 | Vvi-Vitvi17g04185\_t001 |  |  |  |  |  |  |  |  |  |  |  |  |
| 0 | Vvi-Vitvi17g00656\_t001 |  |  |  |  |  |  |  |  |  |  |  |  |
| 0 | Vvi-Vitvi17g00658\_t003 |  |  |  |  |  |  |  |  |  |  |  |  |
| 0 | Vvi-Vitvi17g01485\_t001 |  |  |  |  |  |  |  |  |  |  |  |  |
| 0 | Vvi-Vitvi17g01486\_t001 |  |  |  |  |  |  |  |  |  |  |  |  |
| 0 | Vvi-Vitvi17g00659\_t001 |  |  |  |  |  |  |  |  |  |  |  |  |
| 0 | Vvi-Vitvi17g00660\_t001 |  |  |  |  |  |  |  |  |  |  |  |  |
| 0 | Vvi-Vitvi17g00661\_t001 |  |  |  |  |  |  |  |  |  |  |  |  |
| 0 | Vvi-Vitvi17g04186\_t001 |  |  |  |  |  |  |  |  |  |  |  |  |
| 0 | Vvi-Vitvi17g00663\_t001 |  |  |  |  |  |  |  |  |  |  |  |  |
| 0 | Vvi-Vitvi17g00664\_t001 |  |  |  |  |  |  |  |  |  |  |  |  |
| 0 | Vvi-Vitvi17g04187\_t001 |  |  |  |  |  |  |  |  |  |  |  |  |
| 0 | Vvi-Vitvi17g00665\_t001 |  |  |  |  |  |  |  |  |  |  |  |  |
| 0 | Vvi-Vitvi17g00666\_t001 |  |  |  |  |  |  |  |  |  |  |  |  |
| 1 | Vvi-Vitvi17g00669\_t001 |  | Atr-ERM99921 |  |  |  |  |  |  |  |  |  |  |
| 1 | Vvi-Vitvi17g00670\_t001 |  | Atr-ERM99922 |  |  |  |  |  |  |  |  |  |  |
| 1 | Vvi-Vitvi17g04188\_t001 |  | \| \| |  |  |  |  |  |  |  |  |  |  |
| 1 | Vvi-Vitvi17g01488\_t001 |  | Atr-ERM99927 |  |  |  |  |  |  |  |  |  |  |
| 1 | Vvi-Vitvi17g00671\_t003 |  | Atr-ERM99930 |  |  |  |  |  |  |  |  |  |  |
| 1 | Vvi-Vitvi17g01490\_t001 |  | Atr-ERM99932 |  |  |  |  |  |  |  |  |  |  |
| 1 | Vvi-Vitvi17g04189\_t001 |  | \| \| |  |  |  |  |  |  |  |  |  |  |
| 2 | Vvi-Vitvi17g04190\_t001 |  | \| \| |  | Atr-ERM99899 |  |  |  |  |  |  |  |  |
| 2 | Vvi-Vitvi17g00673\_t001 |  | \| \| |  | Atr-ERM99894 |  |  |  |  |  |  |  |  |
| 2 | Vvi-Vitvi17g00674\_t001 |  | \| \| |  | Atr-ERM99892 |  |  |  |  |  |  |  |  |
| 2 | Vvi-Vitvi17g04191\_t001 |  | \| \| |  | \| \| |  |  |  |  |  |  |  |  |
| 2 | Vvi-Vitvi17g00675\_t001 |  | \| \| |  | Atr-ERM99888 |  |  |  |  |  |  |  |  |
| 2 | Vvi-Vitvi17g00677\_t001 |  | \| \| |  | Atr-ERM99887 |  |  |  |  |  |  |  |  |
| 2 | Vvi-Vitvi17g00678\_t001 |  | \| \| |  | Atr-ERM99884 |  |  |  |  |  |  |  |  |
| 2 | Vvi-Vitvi17g00680\_t001 |  | \| \| |  | Atr-ERM99919 |  |  |  |  |  |  |  |  |
| 2 | Vvi-Vitvi17g00681\_t001 |  | \| \| |  | Atr-ERM99915 |  |  |  |  |  |  |  |  |
| 2 | Vvi-Vitvi17g00682\_t002 |  | \| \| |  | Atr-ERM99914 |  |  |  |  |  |  |  |  |
| 2 | Vvi-Vitvi17g04192\_t001 |  | \| \| |  | Atr-ERM99913 |  |  |  |  |  |  |  |  |
| 2 | Vvi-Vitvi17g00683\_t001 |  | \| \| |  | Atr-ERM99911 |  |  |  |  |  |  |  |  |
| 2 | Vvi-Vitvi17g04193\_t001 |  | \| \| |  | \| \| |  |  |  |  |  |  |  |  |
| 2 | Vvi-Vitvi17g00684\_t001 |  | \| \| |  | Atr-ERM99909 |  |  |  |  |  |  |  |  |
| 1 | Vvi-Vitvi17g00686\_t001 |  | \| \| |  |  |  |  |  |  |  |  |  |  |
| 1 | Vvi-Vitvi17g01492\_t001 |  | \| \| |  |  |  |  |  |  |  |  |  |  |
| 1 | Vvi-Vitvi17g04194\_t001 |  | \| \| |  |  |  |  |  |  |  |  |  |  |
| 1 | Vvi-Vitvi17g04195\_t001 |  | Atr-ERM99950 |  |  |  |  |  |  |  |  |  |  |
| 1 | Vvi-Vitvi17g04196\_t001 |  | \| \| |  |  |  |  |  |  |  |  |  |  |
| 1 | Vvi-Vitvi17g04197\_t001 |  | \| \| |  |  |  |  |  |  |  |  |  |  |
| 1 | Vvi-Vitvi17g01495\_t001 |  | \| \| |  |  |  |  |  |  |  |  |  |  |
| 1 | Vvi-Vitvi17g01496\_t001 |  | \| \| |  |  |  |  |  |  |  |  |  |  |
| 1 | Vvi-Vitvi17g04198\_t001 |  | \| \| |  |  |  |  |  |  |  |  |  |  |
| 1 | Vvi-Vitvi17g01497\_t001 |  | \| \| |  |  |  |  |  |  |  |  |  |  |
| 1 | Vvi-Vitvi17g04199\_t001 |  | \| \| |  |  |  |  |  |  |  |  |  |  |
| 1 | Vvi-Vitvi17g01499\_t001 |  | \| \| |  |  |  |  |  |  |  |  |  |  |
| 1 | Vvi-Vitvi17g04200\_t001 |  | Atr-ERM99953 |  |  |  |  |  |  |  |  |  |  |
| 1 | Vvi-Vitvi17g04201\_t001 |  | \| \| |  |  |  |  |  |  |  |  |  |  |
| 1 | Vvi-Vitvi17g01502\_t001 |  | \| \| |  |  |  |  |  |  |  |  |  |  |
| 1 | Vvi-Vitvi17g00689\_t001 |  | \| \| |  |  |  |  |  |  |  |  |  |  |
| 1 | Vvi-Vitvi17g00690\_t001 |  | Atr-ERM99968 |  |  |  |  |  |  |  |  |  |  |
| 1 | Vvi-Vitvi17g01504\_t001 |  | Atr-ERM99969 |  |  |  |  |  |  |  |  |  |  |
| 1 | Vvi-Vitvi17g01306\_t001 |  | \| \| |  |  |  |  |  |  |  |  |  |  |
| 1 | Vvi-Vitvi17g01505\_t001 |  | \| \| |  |  |  |  |  |  |  |  |  |  |
| 1 | Vvi-Vitvi17g04202\_t001 |  | \| \| |  |  |  |  |  |  |  |  |  |  |
| 1 | Vvi-Vitvi17g00691\_t001 |  | \| \| |  |  |  |  |  |  |  |  |  |  |
| 1 | Vvi-Vitvi17g00692\_t004 |  | Atr-ERM99973 |  |  |  |  |  |  |  |  |  |  |
| 1 | Vvi-Vitvi17g00694\_t001 |  | \| \| |  |  |  |  |  |  |  |  |  |  |
| 1 | Vvi-Vitvi17g01506\_t001 |  | \| \| |  |  |  |  |  |  |  |  |  |  |
| 1 | Vvi-Vitvi17g04203\_t001 |  | \| \| |  |  |  |  |  |  |  |  |  |  |
| 1 | Vvi-Vitvi17g00695\_t001 |  | \| \| |  |  |  |  |  |  |  |  |  |  |
| 1 | Vvi-Vitvi17g00700\_t002 |  | \| \| |  |  |  |  |  |  |  |  |  |  |
| 1 | Vvi-Vitvi17g00701\_t001 |  | \| \| |  |  |  |  |  |  |  |  |  |  |
| 1 | Vvi-Vitvi17g00702\_t001 |  | \| \| |  |  |  |  |  |  |  |  |  |  |
| 1 | Vvi-Vitvi17g04204\_t001 |  | \| \| |  |  |  |  |  |  |  |  |  |  |
| 1 | Vvi-Vitvi17g04205\_t001 |  | \| \| |  |  |  |  |  |  |  |  |  |  |
| 1 | Vvi-Vitvi17g01511\_t001 |  | \| \| |  |  |  |  |  |  |  |  |  |  |
| 1 | Vvi-Vitvi17g04206\_t001 |  | \| \| |  |  |  |  |  |  |  |  |  |  |
| 1 | Vvi-Vitvi17g00704\_t002 |  | \| \| |  |  |  |  |  |  |  |  |  |  |
| 1 | Vvi-Vitvi17g00705\_t001 |  | Atr-ERM99985 |  |  |  |  |  |  |  |  |  |  |
| 0 | Vvi-Vitvi17g00708\_t001 |  |  |  |  |  |  |  |  |  |  |  |  |
| 0 | Vvi-Vitvi17g00709\_t001 |  |  |  |  |  |  |  |  |  |  |  |  |
| 0 | Vvi-Vitvi17g00710\_t002 |  |  |  |  |  |  |  |  |  |  |  |  |
| 0 | Vvi-Vitvi17g04207\_t001 |  |  |  |  |  |  |  |  |  |  |  |  |
| 1 | Vvi-Vitvi17g00711\_t001 |  | Atr-ERN07512 |  |  |  |  |  |  |  |  |  |  |
| 1 | Vvi-Vitvi17g01512\_t001 |  | Atr-ERN07513 |  |  |  |  |  |  |  |  |  |  |
| 1 | Vvi-Vitvi17g00712\_t001 |  | Atr-ERN07517 |  |  |  |  |  |  |  |  |  |  |
| 1 | Vvi-Vitvi17g00713\_t001 |  | Atr-ERN07519 |  |  |  |  |  |  |  |  |  |  |
| 1 | Vvi-Vitvi17g00714\_t003 |  | \| \| |  |  |  |  |  |  |  |  |  |  |
| 1 | Vvi-Vitvi17g04208\_t001 |  | \| \| |  |  |  |  |  |  |  |  |  |  |
| 1 | Vvi-Vitvi17g04209\_t001 |  | \| \| |  |  |  |  |  |  |  |  |  |  |
| 1 | Vvi-Vitvi17g01513\_t001 |  | \| \| |  |  |  |  |  |  |  |  |  |  |
| 1 | Vvi-Vitvi17g04210\_t001 |  | \| \| |  |  |  |  |  |  |  |  |  |  |
| 1 | Vvi-Vitvi17g04211\_t001 |  | \| \| |  |  |  |  |  |  |  |  |  |  |
| 1 | Vvi-Vitvi17g01515\_t001 |  | \| \| |  |  |  |  |  |  |  |  |  |  |
| 1 | Vvi-Vitvi17g04212\_t001 |  | \| \| |  |  |  |  |  |  |  |  |  |  |
| 1 | Vvi-Vitvi17g04213\_t001 |  | \| \| |  |  |  |  |  |  |  |  |  |  |
| 1 | Vvi-Vitvi17g04214\_t001 |  | \| \| |  |  |  |  |  |  |  |  |  |  |
| 1 | Vvi-Vitvi17g04215\_t001 |  | \| \| |  |  |  |  |  |  |  |  |  |  |
| 1 | Vvi-Vitvi17g04216\_t001 |  | \| \| |  |  |  |  |  |  |  |  |  |  |
| 1 | Vvi-Vitvi17g04217\_t001 |  | \| \| |  |  |  |  |  |  |  |  |  |  |
| 1 | Vvi-Vitvi17g01520\_t001 |  | \| \| |  |  |  |  |  |  |  |  |  |  |
| 1 | Vvi-Vitvi17g00719\_t001 |  | Atr-ERN07526 |  |  |  |  |  |  |  |  |  |  |
| 1 | Vvi-Vitvi17g01521\_t001 |  | Atr-ERN07539 |  |  |  |  |  |  |  |  |  |  |
| 0 | Vvi-Vitvi17g00721\_t001 |  |  |  |  |  |  |  |  |  |  |  |  |
| 0 | Vvi-Vitvi17g00722\_t001 |  |  |  |  |  |  |  |  |  |  |  |  |
| 0 | Vvi-Vitvi17g00724\_t001 |  |  |  |  |  |  |  |  |  |  |  |  |
| 0 | Vvi-Vitvi17g00725\_t001 |  |  |  |  |  |  |  |  |  |  |  |  |
| 0 | Vvi-Vitvi17g00726\_t001 |  |  |  |  |  |  |  |  |  |  |  |  |
| 0 | Vvi-Vitvi17g04218\_t001 |  |  |  |  |  |  |  |  |  |  |  |  |
| 0 | Vvi-Vitvi17g00727\_t001 |  |  |  |  |  |  |  |  |  |  |  |  |
| 0 | Vvi-Vitvi17g00728\_t001 |  |  |  |  |  |  |  |  |  |  |  |  |
| 0 | Vvi-Vitvi17g01522\_t001 |  |  |  |  |  |  |  |  |  |  |  |  |
| 0 | Vvi-Vitvi17g00730\_t002 |  |  |  |  |  |  |  |  |  |  |  |  |
| 1 | Vvi-Vitvi17g01523\_t001 |  | Atr-ERM99598 |  |  |  |  |  |  |  |  |  |  |
| 2 | Vvi-Vitvi17g00731\_t001 |  | \| \| |  | Atr-ERN18384 |  |  |  |  |  |  |  |  |
| 2 | Vvi-Vitvi17g00732\_t001 |  | \| \| |  | Atr-ERN18382 |  |  |  |  |  |  |  |  |
| 2 | Vvi-Vitvi17g00733\_t002 |  | \| \| |  | Atr-ERN18380 |  |  |  |  |  |  |  |  |
| 2 | Vvi-Vitvi17g00734\_t001 |  | \| \| |  | Atr-ERN18379 |  |  |  |  |  |  |  |  |
| 2 | Vvi-Vitvi17g04219\_t001 |  | \| \| |  | \| \| |  |  |  |  |  |  |  |  |
| 2 | Vvi-Vitvi17g04220\_t001 |  | \| \| |  | \| \| |  |  |  |  |  |  |  |  |
| 2 | Vvi-Vitvi17g00736\_t002 |  | \| \| |  | Atr-ERN18376 |  |  |  |  |  |  |  |  |
| 2 | Vvi-Vitvi17g00738\_t001 |  | \| \| |  | \| \| |  |  |  |  |  |  |  |  |
| 2 | Vvi-Vitvi17g01525\_t001 |  | \| \| |  | Atr-ERN18355 |  |  |  |  |  |  |  |  |
| 2 | Vvi-Vitvi17g04221\_t001 |  | \| \| |  | \| \| |  |  |  |  |  |  |  |  |
| 2 | Vvi-Vitvi17g04222\_t001 |  | \| \| |  | \| \| |  |  |  |  |  |  |  |  |
| 2 | Vvi-Vitvi17g01527\_t001 |  | \| \| |  | Atr-ERN18353 |  |  |  |  |  |  |  |  |
| 2 | Vvi-Vitvi17g00739\_t001 |  | \| \| |  | Atr-ERN18352 |  |  |  |  |  |  |  |  |
| 1 | Vvi-Vitvi17g01528\_t001 |  | \| \| |  |  |  |  |  |  |  |  |  |  |
| 1 | Vvi-Vitvi17g00740\_t001 |  | \| \| |  |  |  |  |  |  |  |  |  |  |
| 1 | Vvi-Vitvi17g04223\_t001 |  | \| \| |  |  |  |  |  |  |  |  |  |  |
| 1 | Vvi-Vitvi17g00742\_t001 |  | \| \| |  |  |  |  |  |  |  |  |  |  |
| 1 | Vvi-Vitvi17g00743\_t001 |  | \| \| |  |  |  |  |  |  |  |  |  |  |
| 1 | Vvi-Vitvi17g00744\_t001 |  | Atr-ERM99597 |  |  |  |  |  |  |  |  |  |  |
| 1 | Vvi-Vitvi17g01530\_t001 |  | Atr-ERM99596 |  |  |  |  |  |  |  |  |  |  |
| 1 | Vvi-Vitvi17g00747\_t001 |  | \| \| |  |  |  |  |  |  |  |  |  |  |
| 1 | Vvi-Vitvi17g00748\_t001 |  | \| \| |  |  |  |  |  |  |  |  |  |  |
| 1 | Vvi-Vitvi17g00750\_t001 |  | \| \| |  |  |  |  |  |  |  |  |  |  |
| 1 | Vvi-Vitvi17g04224\_t001 |  | \| \| |  |  |  |  |  |  |  |  |  |  |
| 1 | Vvi-Vitvi17g00752\_t001 |  | \| \| |  |  |  |  |  |  |  |  |  |  |
| 1 | Vvi-Vitvi17g00753\_t001 |  | \| \| |  |  |  |  |  |  |  |  |  |  |
| 1 | Vvi-Vitvi17g00754\_t001 |  | Atr-ERM99591 |  |  |  |  |  |  |  |  |  |  |
| 1 | Vvi-Vitvi17g01531\_t001 |  | Atr-ERM99588 |  |  |  |  |  |  |  |  |  |  |
| 1 | Vvi-Vitvi17g00755\_t001 |  | Atr-ERM99572 |  |  |  |  |  |  |  |  |  |  |
| 1 | Vvi-Vitvi17g00756\_t001 |  | \| \| |  |  |  |  |  |  |  |  |  |  |
| 1 | Vvi-Vitvi17g00757\_t001 |  | \| \| |  |  |  |  |  |  |  |  |  |  |
| 1 | Vvi-Vitvi17g00758\_t001 |  | \| \| |  |  |  |  |  |  |  |  |  |  |
| 1 | Vvi-Vitvi17g00759\_t001 |  | Atr-ERM99550 |  |  |  |  |  |  |  |  |  |  |
| 1 | Vvi-Vitvi17g01532\_t003 |  | \| \| |  |  |  |  |  |  |  |  |  |  |
| 1 | Vvi-Vitvi17g00761\_t001 |  | \| \| |  |  |  |  |  |  |  |  |  |  |
| 1 | Vvi-Vitvi17g00762\_t001 |  | \| \| |  |  |  |  |  |  |  |  |  |  |
| 1 | Vvi-Vitvi17g00764\_t001 |  | Atr-ERM99532 |  |  |  |  |  |  |  |  |  |  |
| 1 | Vvi-Vitvi17g00765\_t001 |  | \| \| |  |  |  |  |  |  |  |  |  |  |
| 1 | Vvi-Vitvi17g00767\_t001 |  | Atr-ERM99530 |  |  |  |  |  |  |  |  |  |  |
| 1 | Vvi-Vitvi17g00768\_t001 |  | \| \| |  |  |  |  |  |  |  |  |  |  |
| 1 | Vvi-Vitvi17g00770\_t001 |  | Atr-ERM99527 |  |  |  |  |  |  |  |  |  |  |
| 1 | Vvi-Vitvi17g04225\_t001 |  | Atr-ERM99507 |  |  |  |  |  |  |  |  |  |  |
| 1 | Vvi-Vitvi17g00772\_t003 |  | \| \| |  |  |  |  |  |  |  |  |  |  |
| 1 | Vvi-Vitvi17g00773\_t001 |  | Atr-ERM99505 |  |  |  |  |  |  |  |  |  |  |
| 1 | Vvi-Vitvi17g04226\_t001 |  | \| \| |  |  |  |  |  |  |  |  |  |  |
| 1 | Vvi-Vitvi17g00775\_t001 |  | Atr-ERM99504 |  |  |  |  |  |  |  |  |  |  |
| 1 | Vvi-Vitvi17g00776\_t001 |  | Atr-ERM99496 |  |  |  |  |  |  |  |  |  |  |
| 1 | Vvi-Vitvi17g01535\_t001 |  | \| \| |  |  |  |  |  |  |  |  |  |  |
| 1 | Vvi-Vitvi17g00777\_t001 |  | Atr-ERM99494 |  |  |  |  |  |  |  |  |  |  |
| 1 | Vvi-Vitvi17g00778\_t001 |  | Atr-ERN05925 |  |  |  |  |  |  |  |  |  |  |
| 1 | Vvi-Vitvi17g04227\_t001 |  | \| \| |  |  |  |  |  |  |  |  |  |  |
| 1 | Vvi-Vitvi17g00779\_t001 |  | Atr-ERN05923 |  |  |  |  |  |  |  |  |  |  |
| 1 | Vvi-Vitvi17g00780\_t001 |  | Atr-ERN05918 |  |  |  |  |  |  |  |  |  |  |
| 1 | Vvi-Vitvi17g00781\_t001 |  | Atr-ERN05917 |  |  |  |  |  |  |  |  |  |  |
| 1 | Vvi-Vitvi17g04228\_t001 |  | Atr-ERN05914 |  |  |  |  |  |  |  |  |  |  |
| 1 | Vvi-Vitvi17g04229\_t001 |  | \| \| |  |  |  |  |  |  |  |  |  |  |
| 1 | Vvi-Vitvi17g00783\_t001 |  | \| \| |  |  |  |  |  |  |  |  |  |  |
| 1 | Vvi-Vitvi17g00786\_t001 |  | Atr-ERN05911 |  |  |  |  |  |  |  |  |  |  |
| 1 | Vvi-Vitvi17g00787\_t001 |  | Atr-ERN05910 |  |  |  |  |  |  |  |  |  |  |
| 1 | Vvi-Vitvi17g04230\_t001 |  | \| \| |  |  |  |  |  |  |  |  |  |  |
| 1 | Vvi-Vitvi17g00788\_t001 |  | \| \| |  |  |  |  |  |  |  |  |  |  |
| 1 | Vvi-Vitvi17g00789\_t001 |  | \| \| |  |  |  |  |  |  |  |  |  |  |
| 1 | Vvi-Vitvi17g00790\_t001 |  | Atr-ERN05907 |  |  |  |  |  |  |  |  |  |  |
| 1 | Vvi-Vitvi17g00791\_t001 |  | Atr-ERN06624 |  |  |  |  |  |  |  |  |  |  |
| 1 | Vvi-Vitvi17g00793\_t001 |  | \| \| |  |  |  |  |  |  |  |  |  |  |
| 1 | Vvi-Vitvi17g00794\_t001 |  | Atr-ERN06627 |  |  |  |  |  |  |  |  |  |  |
| 1 | Vvi-Vitvi17g00795\_t001 |  | \| \| |  |  |  |  |  |  |  |  |  |  |
| 1 | Vvi-Vitvi17g00796\_t001 |  | Atr-ERN06635 |  |  |  |  |  |  |  |  |  |  |
| 1 | Vvi-Vitvi17g00797\_t001 |  | Atr-ERN06639 |  |  |  |  |  |  |  |  |  |  |
| 1 | Vvi-Vitvi17g00799\_t001 |  | Atr-ERN06640 |  |  |  |  |  |  |  |  |  |  |
| 1 | Vvi-Vitvi17g04231\_t001 |  | \| \| |  |  |  |  |  |  |  |  |  |  |
| 1 | Vvi-Vitvi17g00801\_t001 |  | Atr-ERN06644 |  |  |  |  |  |  |  |  |  |  |
| 1 | Vvi-Vitvi17g04232\_t001 |  | \| \| |  |  |  |  |  |  |  |  |  |  |
| 1 | Vvi-Vitvi17g00802\_t001 |  | Atr-ERN06645 |  |  |  |  |  |  |  |  |  |  |
| 1 | Vvi-Vitvi17g00803\_t001 |  | Atr-ERN06646 |  |  |  |  |  |  |  |  |  |  |
| 1 | Vvi-Vitvi17g00804\_t001 |  | Atr-ERN06651 |  |  |  |  |  |  |  |  |  |  |
| 1 | Vvi-Vitvi17g00805\_t001 |  | \| \| |  |  |  |  |  |  |  |  |  |  |
| 1 | Vvi-Vitvi17g00807\_t002 |  | Atr-ERN06655 |  |  |  |  |  |  |  |  |  |  |
| 1 | Vvi-Vitvi17g00808\_t001 |  | \| \| |  |  |  |  |  |  |  |  |  |  |
| 2 | Vvi-Vitvi17g00809\_t001 |  | \| \| |  | Atr-ERN06694 |  |  |  |  |  |  |  |  |
| 2 | Vvi-Vitvi17g04233\_t001 |  | \| \| |  | \| \| |  |  |  |  |  |  |  |  |
| 2 | Vvi-Vitvi17g04234\_t001 |  | \| \| |  | \| \| |  |  |  |  |  |  |  |  |
| 2 | Vvi-Vitvi17g04235\_t001 |  | \| \| |  | \| \| |  |  |  |  |  |  |  |  |
| 2 | Vvi-Vitvi17g04236\_t001 |  | \| \| |  | \| \| |  |  |  |  |  |  |  |  |
| 2 | Vvi-Vitvi17g00813\_t001 |  | \| \| |  | Atr-ERN06689 |  |  |  |  |  |  |  |  |
| 2 | Vvi-Vitvi17g00814\_t001 |  | \| \| |  | Atr-ERN06674 |  |  |  |  |  |  |  |  |
| 2 | Vvi-Vitvi17g00815\_t001 |  | \| \| |  | Atr-ERN06668 |  |  |  |  |  |  |  |  |
| 2 | Vvi-Vitvi17g00816\_t001 |  | \| \| |  | Atr-ERN06667 |  |  |  |  |  |  |  |  |
| 2 | Vvi-Vitvi17g01543\_t001 |  | \| \| |  | \| \| |  |  |  |  |  |  |  |  |
| 2 | Vvi-Vitvi17g00817\_t001 |  | \| \| |  | Atr-ERN06664 |  |  |  |  |  |  |  |  |
| 1 | Vvi-Vitvi17g00818\_t001 |  | Atr-ERN06656 |  |  |  |  |  |  |  |  |  |  |
| 1 | Vvi-Vitvi17g04237\_t001 |  | \| \| |  |  |  |  |  |  |  |  |  |  |
| 1 | Vvi-Vitvi17g04238\_t001 |  | \| \| |  |  |  |  |  |  |  |  |  |  |
| 1 | Vvi-Vitvi17g01307\_t001 |  | \| \| |  |  |  |  |  |  |  |  |  |  |
| 1 | Vvi-Vitvi17g00819\_t001 |  | Atr-ERN06663 |  |  |  |  |  |  |  |  |  |  |
| 0 | Vvi-Vitvi17g00820\_t001 |  |  |  |  |  |  |  |  |  |  |  |  |
| 0 | Vvi-Vitvi17g00821\_t001 |  |  |  |  |  |  |  |  |  |  |  |  |
| 0 | Vvi-Vitvi17g00822\_t001 |  |  |  |  |  |  |  |  |  |  |  |  |
| 0 | Vvi-Vitvi17g00823\_t002 |  |  |  |  |  |  |  |  |  |  |  |  |
| 0 | Vvi-Vitvi17g00824\_t001 |  |  |  |  |  |  |  |  |  |  |  |  |
| 0 | Vvi-Vitvi17g00825\_t001 |  |  |  |  |  |  |  |  |  |  |  |  |
| 0 | Vvi-Vitvi17g00826\_t001 |  |  |  |  |  |  |  |  |  |  |  |  |
| 1 | Vvi-Vitvi17g00829\_t001 |  | Atr-ERN02375 |  |  |  |  |  |  |  |  |  |  |
| 1 | Vvi-Vitvi17g00831\_t001 |  | Atr-ERN02367 |  |  |  |  |  |  |  |  |  |  |
| 1 | Vvi-Vitvi17g00832\_t001 |  | Atr-ERN02363 |  |  |  |  |  |  |  |  |  |  |
| 1 | Vvi-Vitvi17g00833\_t001 |  | Atr-ERN02362 |  |  |  |  |  |  |  |  |  |  |
| 1 | Vvi-Vitvi17g00834\_t001 |  | Atr-ERN02360 |  |  |  |  |  |  |  |  |  |  |
| 1 | Vvi-Vitvi17g04239\_t001 |  | \| \| |  |  |  |  |  |  |  |  |  |  |
| 1 | Vvi-Vitvi17g00836\_t001 |  | Atr-ERN02352 |  |  |  |  |  |  |  |  |  |  |
| 1 | Vvi-Vitvi17g00837\_t003 |  | Atr-ERN02351 |  |  |  |  |  |  |  |  |  |  |
| 1 | Vvi-Vitvi17g00838\_t001 |  | \| \| |  |  |  |  |  |  |  |  |  |  |
| 1 | Vvi-Vitvi17g00839\_t002 |  | Atr-ERN02350 |  |  |  |  |  |  |  |  |  |  |
| 1 | Vvi-Vitvi17g00840\_t001 |  | Atr-ERN02348 |  |  |  |  |  |  |  |  |  |  |
| 1 | Vvi-Vitvi17g00841\_t001 |  | Atr-ERN02347 |  |  |  |  |  |  |  |  |  |  |
| 1 | Vvi-Vitvi17g00842\_t001 |  | Atr-ERN02345 |  |  |  |  |  |  |  |  |  |  |
| 1 | Vvi-Vitvi17g00843\_t001 |  | Atr-ERN02339 |  |  |  |  |  |  |  |  |  |  |
| 1 | Vvi-Vitvi17g00844\_t001 |  | Atr-ERN02338 |  |  |  |  |  |  |  |  |  |  |
| 1 | Vvi-Vitvi17g00845\_t001 |  | Atr-ERN02335 |  |  |  |  |  |  |  |  |  |  |
| 0 | Vvi-Vitvi17g00848\_t001 |  |  |  |  |  |  |  |  |  |  |  |  |
| 0 | Vvi-Vitvi17g01550\_t001 |  |  |  |  |  |  |  |  |  |  |  |  |
| 0 | Vvi-Vitvi17g04240\_t001 |  |  |  |  |  |  |  |  |  |  |  |  |
| 0 | Vvi-Vitvi17g04241\_t001 |  |  |  |  |  |  |  |  |  |  |  |  |
| 0 | Vvi-Vitvi17g04242\_t001 |  |  |  |  |  |  |  |  |  |  |  |  |
| 1 | Vvi-Vitvi17g00850\_t001 |  | Atr-ERN02729 |  |  |  |  |  |  |  |  |  |  |
| 1 | Vvi-Vitvi17g00853\_t001 |  | \| \| |  |  |  |  |  |  |  |  |  |  |
| 1 | Vvi-Vitvi17g00854\_t001 |  | Atr-ERN02723 |  |  |  |  |  |  |  |  |  |  |
| 1 | Vvi-Vitvi17g04243\_t001 |  | \| \| |  |  |  |  |  |  |  |  |  |  |
| 1 | Vvi-Vitvi17g04244\_t001 |  | \| \| |  |  |  |  |  |  |  |  |  |  |
| 1 | Vvi-Vitvi17g00856\_t002 |  | Atr-ERN02722 |  |  |  |  |  |  |  |  |  |  |
| 1 | Vvi-Vitvi17g04245\_t001 |  | \| \| |  |  |  |  |  |  |  |  |  |  |
| 1 | Vvi-Vitvi17g00861\_t002 |  | Atr-ERN02717 |  |  |  |  |  |  |  |  |  |  |
| 1 | Vvi-Vitvi17g00862\_t001 |  | Atr-ERN02699 |  |  |  |  |  |  |  |  |  |  |
| 1 | Vvi-Vitvi17g00863\_t001 |  | Atr-ERN02698 |  |  |  |  |  |  |  |  |  |  |
| 1 | Vvi-Vitvi17g00864\_t001 |  | Atr-ERN02696 |  |  |  |  |  |  |  |  |  |  |
| 1 | Vvi-Vitvi17g00866\_t001 |  | \| \| |  |  |  |  |  |  |  |  |  |  |
| 1 | Vvi-Vitvi17g04246\_t001 |  | \| \| |  |  |  |  |  |  |  |  |  |  |
| 1 | Vvi-Vitvi17g00867\_t001 |  | \| \| |  |  |  |  |  |  |  |  |  |  |
| 1 | Vvi-Vitvi17g04247\_t001 |  | \| \| |  |  |  |  |  |  |  |  |  |  |
| 1 | Vvi-Vitvi17g04248\_t001 |  | \| \| |  |  |  |  |  |  |  |  |  |  |
| 1 | Vvi-Vitvi17g00869\_t001 |  | Atr-ERN02686 |  |  |  |  |  |  |  |  |  |  |
| 1 | Vvi-Vitvi17g00870\_t001 |  | Atr-ERN02673 |  |  |  |  |  |  |  |  |  |  |
| 1 | Vvi-Vitvi17g04249\_t001 |  | \| \| |  |  |  |  |  |  |  |  |  |  |
| 1 | Vvi-Vitvi17g04250\_t001 |  | \| \| |  |  |  |  |  |  |  |  |  |  |
| 1 | Vvi-Vitvi17g01553\_t001 |  | Atr-ERN02670 |  |  |  |  |  |  |  |  |  |  |
| 1 | Vvi-Vitvi17g00872\_t001 |  | Atr-ERN02666 |  |  |  |  |  |  |  |  |  |  |
| 1 | Vvi-Vitvi17g00873\_t001 |  | \| \| |  |  |  |  |  |  |  |  |  |  |
| 1 | Vvi-Vitvi17g00874\_t001 |  | Atr-ERN02657 |  |  |  |  |  |  |  |  |  |  |
| 1 | Vvi-Vitvi17g00875\_t001 |  | Atr-ERN02655 |  |  |  |  |  |  |  |  |  |  |
| 1 | Vvi-Vitvi17g04251\_t001 |  | \| \| |  |  |  |  |  |  |  |  |  |  |
| 1 | Vvi-Vitvi17g00876\_t001 |  | \| \| |  |  |  |  |  |  |  |  |  |  |
| 1 | Vvi-Vitvi17g00877\_t001 |  | \| \| |  |  |  |  |  |  |  |  |  |  |
| 1 | Vvi-Vitvi17g00878\_t001 |  | \| \| |  |  |  |  |  |  |  |  |  |  |
| 1 | Vvi-Vitvi17g00880\_t001 |  | Atr-ERN02653 |  |  |  |  |  |  |  |  |  |  |
| 1 | Vvi-Vitvi17g00881\_t001 |  | Atr-ERN02651 |  |  |  |  |  |  |  |  |  |  |
| 1 | Vvi-Vitvi17g00882\_t001 |  | \| \| |  |  |  |  |  |  |  |  |  |  |
| 1 | Vvi-Vitvi17g00883\_t001 |  | Atr-ERN02648 |  |  |  |  |  |  |  |  |  |  |
| 1 | Vvi-Vitvi17g00884\_t001 |  | Atr-ERN02643 |  |  |  |  |  |  |  |  |  |  |
| 1 | Vvi-Vitvi17g00885\_t001 |  | Atr-ERN02642 |  |  |  |  |  |  |  |  |  |  |
| 0 | Vvi-Vitvi17g00886\_t001 |  |  |  |  |  |  |  |  |  |  |  |  |
| 0 | Vvi-Vitvi17g04252\_t001 |  |  |  |  |  |  |  |  |  |  |  |  |
| 0 | Vvi-Vitvi17g00888\_t001 |  |  |  |  |  |  |  |  |  |  |  |  |
| 0 | Vvi-Vitvi17g00889\_t001 |  |  |  |  |  |  |  |  |  |  |  |  |
| 0 | Vvi-Vitvi17g01554\_t001 |  |  |  |  |  |  |  |  |  |  |  |  |
| 0 | Vvi-Vitvi17g00890\_t002 |  |  |  |  |  |  |  |  |  |  |  |  |
| 0 | Vvi-Vitvi17g00891\_t001 |  |  |  |  |  |  |  |  |  |  |  |  |
| 0 | Vvi-Vitvi17g01555\_t001 |  |  |  |  |  |  |  |  |  |  |  |  |
| 0 | Vvi-Vitvi17g00892\_t001 |  |  |  |  |  |  |  |  |  |  |  |  |
| 0 | Vvi-Vitvi17g01556\_t001 |  |  |  |  |  |  |  |  |  |  |  |  |
| 0 | Vvi-Vitvi17g04253\_t001 |  |  |  |  |  |  |  |  |  |  |  |  |
| 0 | Vvi-Vitvi17g00893\_t001 |  |  |  |  |  |  |  |  |  |  |  |  |
| 0 | Vvi-Vitvi17g00894\_t001 |  |  |  |  |  |  |  |  |  |  |  |  |
| 0 | Vvi-Vitvi17g00895\_t001 |  |  |  |  |  |  |  |  |  |  |  |  |
| 0 | Vvi-Vitvi17g00897\_t001 |  |  |  |  |  |  |  |  |  |  |  |  |
| 0 | Vvi-Vitvi17g00898\_t001 |  |  |  |  |  |  |  |  |  |  |  |  |
| 0 | Vvi-Vitvi17g04254\_t001 |  |  |  |  |  |  |  |  |  |  |  |  |
| 0 | Vvi-Vitvi17g00899\_t001 |  |  |  |  |  |  |  |  |  |  |  |  |
| 0 | Vvi-Vitvi17g00900\_t001 |  |  |  |  |  |  |  |  |  |  |  |  |
| 0 | Vvi-Vitvi17g00901\_t001 |  |  |  |  |  |  |  |  |  |  |  |  |
| 1 | Vvi-Vitvi17g00903\_t001 |  | Atr-ERN13778 |  |  |  |  |  |  |  |  |  |  |
| 1 | Vvi-Vitvi17g00904\_t001 |  | \| \| |  |  |  |  |  |  |  |  |  |  |
| 1 | Vvi-Vitvi17g00905\_t001 |  | Atr-ERN13792 |  |  |  |  |  |  |  |  |  |  |
| 1 | Vvi-Vitvi17g04255\_t001 |  | \| \| |  |  |  |  |  |  |  |  |  |  |
| 1 | Vvi-Vitvi17g00907\_t001 |  | Atr-ERN13798 |  |  |  |  |  |  |  |  |  |  |
| 2 | Vvi-Vitvi17g00909\_t001 |  | Atr-ERN13799 |  | Atr-ERN19192 |  |  |  |  |  |  |  |  |
| 2 | Vvi-Vitvi17g00910\_t001 |  | Atr-ERN13801 |  | \| \| |  |  |  |  |  |  |  |  |
| 2 | Vvi-Vitvi17g00911\_t001 |  | Atr-ERN13802 |  | \| \| |  |  |  |  |  |  |  |  |
| 2 | Vvi-Vitvi17g04256\_t001 |  | \| \| |  | \| \| |  |  |  |  |  |  |  |  |
| 2 | Vvi-Vitvi17g00915\_t001 |  | \| \| |  | \| \| |  |  |  |  |  |  |  |  |
| 2 | Vvi-Vitvi17g04257\_t001 |  | \| \| |  | \| \| |  |  |  |  |  |  |  |  |
| 2 | Vvi-Vitvi17g00916\_t003 |  | Atr-ERN13808 |  | \| \| |  |  |  |  |  |  |  |  |
| 2 | Vvi-Vitvi17g00917\_t001 |  | Atr-ERN13816 |  | \| \| |  |  |  |  |  |  |  |  |
| 2 | Vvi-Vitvi17g00918\_t001 |  | \| \| |  | \| \| |  |  |  |  |  |  |  |  |
| 2 | Vvi-Vitvi17g04258\_t001 |  | \| \| |  | \| \| |  |  |  |  |  |  |  |  |
| 2 | Vvi-Vitvi17g04259\_t001 |  | Atr-ERN13832 |  | \| \| |  |  |  |  |  |  |  |  |
| 2 | Vvi-Vitvi17g00920\_t001 |  | Atr-ERN13833 |  | \| \| |  |  |  |  |  |  |  |  |
| 2 | Vvi-Vitvi17g00921\_t001 |  | Atr-ERN13836 |  | Atr-ERN19176 |  |  |  |  |  |  |  |  |
| 2 | Vvi-Vitvi17g04260\_t001 |  | \| \| |  | \| \| |  |  |  |  |  |  |  |  |
| 2 | Vvi-Vitvi17g04261\_t001 |  | \| \| |  | \| \| |  |  |  |  |  |  |  |  |
| 2 | Vvi-Vitvi17g04262\_t001 |  | \| \| |  | \| \| |  |  |  |  |  |  |  |  |
| 2 | Vvi-Vitvi17g00927\_t001 |  | Atr-ERN13838 |  | \| \| |  |  |  |  |  |  |  |  |
| 2 | Vvi-Vitvi17g00928\_t001 |  | Atr-ERN13845 |  | \| \| |  |  |  |  |  |  |  |  |
| 2 | Vvi-Vitvi17g00929\_t002 |  | Atr-ERN13846 |  | \| \| |  |  |  |  |  |  |  |  |
| 2 | Vvi-Vitvi17g00930\_t001 |  | \| \| |  | \| \| |  |  |  |  |  |  |  |  |
| 2 | Vvi-Vitvi17g00931\_t001 |  | \| \| |  | Atr-ERN19165 |  |  |  |  |  |  |  |  |
| 2 | Vvi-Vitvi17g00932\_t001 |  | Atr-ERN13848 |  | \| \| |  |  |  |  |  |  |  |  |
| 2 | Vvi-Vitvi17g04263\_t001 |  | \| \| |  | \| \| |  |  |  |  |  |  |  |  |
| 2 | Vvi-Vitvi17g04264\_t001 |  | \| \| |  | \| \| |  |  |  |  |  |  |  |  |
| 2 | Vvi-Vitvi17g01564\_t001 |  | \| \| |  | \| \| |  |  |  |  |  |  |  |  |
| 2 | Vvi-Vitvi17g04265\_t001 |  | \| \| |  | \| \| |  |  |  |  |  |  |  |  |
| 2 | Vvi-Vitvi17g04266\_t001 |  | \| \| |  | \| \| |  |  |  |  |  |  |  |  |
| 2 | Vvi-Vitvi17g04267\_t001 |  | \| \| |  | \| \| |  |  |  |  |  |  |  |  |
| 2 | Vvi-Vitvi17g04268\_t001 |  | \| \| |  | \| \| |  |  |  |  |  |  |  |  |
| 2 | Vvi-Vitvi17g00936\_t001 |  | \| \| |  | \| \| |  |  |  |  |  |  |  |  |
| 2 | Vvi-Vitvi17g00937\_t001 |  | \| \| |  | \| \| |  |  |  |  |  |  |  |  |
| 2 | Vvi-Vitvi17g00938\_t001 |  | \| \| |  | Atr-ERN19148 |  |  |  |  |  |  |  |  |
| 2 | Vvi-Vitvi17g00941\_t001 |  | Atr-ERN13858 |  | \| \| |  |  |  |  |  |  |  |  |
| 1 | Vvi-Vitvi17g00942\_t001 |  |  |  | Atr-ERN19140 |  |  |  |  |  |  |  |  |
| 1 | Vvi-Vitvi17g00944\_t001 |  |  |  | \| \| |  |  |  |  |  |  |  |  |
| 1 | Vvi-Vitvi17g04269\_t001 |  |  |  | \| \| |  |  |  |  |  |  |  |  |
| 1 | Vvi-Vitvi17g01571\_t001 |  |  |  | \| \| |  |  |  |  |  |  |  |  |
| 1 | Vvi-Vitvi17g01574\_t001 |  |  |  | \| \| |  |  |  |  |  |  |  |  |
| 1 | Vvi-Vitvi17g00952\_t001 |  |  |  | \| \| |  |  |  |  |  |  |  |  |
| 1 | Vvi-Vitvi17g01576\_t001 |  |  |  | \| \| |  |  |  |  |  |  |  |  |
| 2 | Vvi-Vitvi17g00953\_t001 |  | Atr-ERN10547 |  | \| \| |  |  |  |  |  |  |  |  |
| 2 | Vvi-Vitvi17g04270\_t001 |  | \| \| |  | \| \| |  |  |  |  |  |  |  |  |
| 2 | Vvi-Vitvi17g01577\_t001 |  | \| \| |  | \| \| |  |  |  |  |  |  |  |  |
| 2 | Vvi-Vitvi17g00954\_t001 |  | Atr-ERN10544 |  | Atr-ERN19128 |  |  |  |  |  |  |  |  |
| 2 | Vvi-Vitvi17g00955\_t001 |  | Atr-ERN10543 |  | \| \| |  |  |  |  |  |  |  |  |
| 2 | Vvi-Vitvi17g04271\_t001 |  | Atr-ERN10542 |  | \| \| |  |  |  |  |  |  |  |  |
| 2 | Vvi-Vitvi17g04272\_t001 |  | \| \| |  | \| \| |  |  |  |  |  |  |  |  |
| 2 | Vvi-Vitvi17g00957\_t001 |  | \| \| |  | \| \| |  |  |  |  |  |  |  |  |
| 2 | Vvi-Vitvi17g00958\_t001 |  | Atr-ERN10538 |  | Atr-ERN19114 |  |  |  |  |  |  |  |  |
| 1 | Vvi-Vitvi17g00959\_t001 |  | Atr-ERN10516 |  |  |  |  |  |  |  |  |  |  |
| 1 | Vvi-Vitvi17g00963\_t001 |  | Atr-ERN10511 |  |  |  |  |  |  |  |  |  |  |
| 1 | Vvi-Vitvi17g00964\_t001 |  | Atr-ERN10509 |  |  |  |  |  |  |  |  |  |  |
| 1 | Vvi-Vitvi17g01581\_t001 |  | Atr-ERN10508 |  |  |  |  |  |  |  |  |  |  |
| 0 | Vvi-Vitvi17g04273\_t001 |  |  |  |  |  |  |  |  |  |  |  |  |
| 1 | Vvi-Vitvi17g00968\_t001 |  | Atr-ERN13671 |  |  |  |  |  |  |  |  |  |  |
| 1 | Vvi-Vitvi17g00969\_t001 |  | Atr-ERN13672 |  |  |  |  |  |  |  |  |  |  |
| 1 | Vvi-Vitvi17g00970\_t001 |  | \| \| |  |  |  |  |  |  |  |  |  |  |
| 1 | Vvi-Vitvi17g00971\_t001 |  | \| \| |  |  |  |  |  |  |  |  |  |  |
| 1 | Vvi-Vitvi17g00972\_t001 |  | \| \| |  |  |  |  |  |  |  |  |  |  |
| 1 | Vvi-Vitvi17g00973\_t001 |  | Atr-ERN13683 |  |  |  |  |  |  |  |  |  |  |
| 1 | Vvi-Vitvi17g00974\_t001 |  | \| \| |  |  |  |  |  |  |  |  |  |  |
| 1 | Vvi-Vitvi17g00975\_t001 |  | Atr-ERN13690 |  |  |  |  |  |  |  |  |  |  |
| 1 | Vvi-Vitvi17g00977\_t001 |  | \| \| |  |  |  |  |  |  |  |  |  |  |
| 1 | Vvi-Vitvi17g04274\_t001 |  | \| \| |  |  |  |  |  |  |  |  |  |  |
| 1 | Vvi-Vitvi17g00979\_t001 |  | \| \| |  |  |  |  |  |  |  |  |  |  |
| 1 | Vvi-Vitvi17g04275\_t001 |  | \| \| |  |  |  |  |  |  |  |  |  |  |
| 1 | Vvi-Vitvi17g00983\_t001 |  | \| \| |  |  |  |  |  |  |  |  |  |  |
| 1 | Vvi-Vitvi17g00985\_t001 |  | \| \| |  |  |  |  |  |  |  |  |  |  |
| 1 | Vvi-Vitvi17g00986\_t001 |  | \| \| |  |  |  |  |  |  |  |  |  |  |
| 1 | Vvi-Vitvi17g04276\_t001 |  | \| \| |  |  |  |  |  |  |  |  |  |  |
| 1 | Vvi-Vitvi17g00987\_t001 |  | \| \| |  |  |  |  |  |  |  |  |  |  |
| 1 | Vvi-Vitvi17g04277\_t001 |  | \| \| |  |  |  |  |  |  |  |  |  |  |
| 1 | Vvi-Vitvi17g04278\_t001 |  | \| \| |  |  |  |  |  |  |  |  |  |  |
| 1 | Vvi-Vitvi17g04279\_t001 |  | \| \| |  |  |  |  |  |  |  |  |  |  |
| 1 | Vvi-Vitvi17g04280\_t001 |  | \| \| |  |  |  |  |  |  |  |  |  |  |
| 1 | Vvi-Vitvi17g00989\_t001 |  | Atr-ERN13713 |  |  |  |  |  |  |  |  |  |  |
| 1 | Vvi-Vitvi17g00990\_t001 |  | \| \| |  |  |  |  |  |  |  |  |  |  |
| 1 | Vvi-Vitvi17g01585\_t001 |  | Atr-ERN13738 |  |  |  |  |  |  |  |  |  |  |
| 1 | Vvi-Vitvi17g00991\_t001 |  | \| \| |  |  |  |  |  |  |  |  |  |  |
| 1 | Vvi-Vitvi17g00994\_t001 |  | \| \| |  |  |  |  |  |  |  |  |  |  |
| 1 | Vvi-Vitvi17g00995\_t001 |  | \| \| |  |  |  |  |  |  |  |  |  |  |
| 1 | Vvi-Vitvi17g00996\_t001 |  | \| \| |  |  |  |  |  |  |  |  |  |  |
| 1 | Vvi-Vitvi17g00998\_t001 |  | \| \| |  |  |  |  |  |  |  |  |  |  |
| 1 | Vvi-Vitvi17g01000\_t001 |  | \| \| |  |  |  |  |  |  |  |  |  |  |
| 1 | Vvi-Vitvi17g01001\_t001 |  | Atr-ERN13745 |  |  |  |  |  |  |  |  |  |  |
| 0 | Vvi-Vitvi17g01002\_t001 |  |  |  |  |  |  |  |  |  |  |  |  |
| 0 | Vvi-Vitvi17g01003\_t001 |  |  |  |  |  |  |  |  |  |  |  |  |
| 0 | Vvi-Vitvi17g01006\_t001 |  |  |  |  |  |  |  |  |  |  |  |  |
| 0 | Vvi-Vitvi17g01011\_t004 |  |  |  |  |  |  |  |  |  |  |  |  |
| 0 | Vvi-Vitvi17g01012\_t001 |  |  |  |  |  |  |  |  |  |  |  |  |
| 1 | Vvi-Vitvi17g01013\_t002 |  | Atr-ERN13629 |  |  |  |  |  |  |  |  |  |  |
| 1 | Vvi-Vitvi17g04281\_t001 |  | \| \| |  |  |  |  |  |  |  |  |  |  |
| 1 | Vvi-Vitvi17g04282\_t001 |  | \| \| |  |  |  |  |  |  |  |  |  |  |
| 1 | Vvi-Vitvi17g01015\_t001 |  | \| \| |  |  |  |  |  |  |  |  |  |  |
| 1 | Vvi-Vitvi17g01016\_t001 |  | \| \| |  |  |  |  |  |  |  |  |  |  |
| 1 | Vvi-Vitvi17g01017\_t001 |  | \| \| |  |  |  |  |  |  |  |  |  |  |
| 1 | Vvi-Vitvi17g01018\_t001 |  | \| \| |  |  |  |  |  |  |  |  |  |  |
| 1 | Vvi-Vitvi17g04283\_t001 |  | \| \| |  |  |  |  |  |  |  |  |  |  |
| 1 | Vvi-Vitvi17g01021\_t001 |  | Atr-ERN13624 |  |  |  |  |  |  |  |  |  |  |
| 1 | Vvi-Vitvi17g01024\_t001 |  | \| \| |  |  |  |  |  |  |  |  |  |  |
| 1 | Vvi-Vitvi17g01025\_t001 |  | \| \| |  |  |  |  |  |  |  |  |  |  |
| 1 | Vvi-Vitvi17g01026\_t001 |  | \| \| |  |  |  |  |  |  |  |  |  |  |
| 1 | Vvi-Vitvi17g01030\_t001 |  | \| \| |  |  |  |  |  |  |  |  |  |  |
| 1 | Vvi-Vitvi17g01034\_t001 |  | \| \| |  |  |  |  |  |  |  |  |  |  |
| 1 | Vvi-Vitvi17g01036\_t001 |  | \| \| |  |  |  |  |  |  |  |  |  |  |
| 1 | Vvi-Vitvi17g01037\_t002 |  | Atr-ERN13619 |  |  |  |  |  |  |  |  |  |  |
| 1 | Vvi-Vitvi17g01592\_t001 |  | Atr-ERN13607 |  |  |  |  |  |  |  |  |  |  |
| 1 | Vvi-Vitvi17g01593\_t001 |  | \| \| |  |  |  |  |  |  |  |  |  |  |
| 1 | Vvi-Vitvi17g01038\_t002 |  | Atr-ERN13605 |  |  |  |  |  |  |  |  |  |  |
| 1 | Vvi-Vitvi17g01039\_t001 |  | \| \| |  |  |  |  |  |  |  |  |  |  |
| 1 | Vvi-Vitvi17g01040\_t001 |  | Atr-ERN13603 |  |  |  |  |  |  |  |  |  |  |
| 0 | Vvi-Vitvi17g04284\_t001 |  |  |  |  |  |  |  |  |  |  |  |  |
| 0 | Vvi-Vitvi17g01596\_t001 |  |  |  |  |  |  |  |  |  |  |  |  |
| 0 | Vvi-Vitvi17g01050\_t001 |  |  |  |  |  |  |  |  |  |  |  |  |
| 0 | Vvi-Vitvi17g01598\_t002 |  |  |  |  |  |  |  |  |  |  |  |  |
| 0 | Vvi-Vitvi17g01275\_t001 |  |  |  |  |  |  |  |  |  |  |  |  |
| 0 | Vvi-Vitvi17g04286\_t001 |  |  |  |  |  |  |  |  |  |  |  |  |
| 0 | Vvi-Vitvi17g01308\_t001 |  |  |  |  |  |  |  |  |  |  |  |  |
| 0 | Vvi-Vitvi17g01278\_t001 |  |  |  |  |  |  |  |  |  |  |  |  |
| 0 | Vvi-Vitvi17g01279\_t001 |  |  |  |  |  |  |  |  |  |  |  |  |
| 0 | Vvi-Vitvi17g04287\_t001 |  |  |  |  |  |  |  |  |  |  |  |  |
| 0 | Vvi-Vitvi17g04288\_t001 |  |  |  |  |  |  |  |  |  |  |  |  |
| 0 | Vvi-Vitvi17g01604\_t001 |  |  |  |  |  |  |  |  |  |  |  |  |
| 0 | Vvi-Vitvi17g04289\_t001 |  |  |  |  |  |  |  |  |  |  |  |  |
| 0 | Vvi-Vitvi17g01068\_t001 |  |  |  |  |  |  |  |  |  |  |  |  |
| 0 | Vvi-Vitvi17g04290\_t001 |  |  |  |  |  |  |  |  |  |  |  |  |
| 0 | Vvi-Vitvi17g04291\_t001 |  |  |  |  |  |  |  |  |  |  |  |  |
| 0 | Vvi-Vitvi17g01605\_t001 |  |  |  |  |  |  |  |  |  |  |  |  |
| 0 | Vvi-Vitvi17g01071\_t001 |  |  |  |  |  |  |  |  |  |  |  |  |
| 0 | Vvi-Vitvi17g01072\_t001 |  |  |  |  |  |  |  |  |  |  |  |  |
| 0 | Vvi-Vitvi17g01073\_t001 |  |  |  |  |  |  |  |  |  |  |  |  |
| 0 | Vvi-Vitvi17g01074\_t001 |  |  |  |  |  |  |  |  |  |  |  |  |
| 0 | Vvi-Vitvi17g04292\_t001 |  |  |  |  |  |  |  |  |  |  |  |  |
| 0 | Vvi-Vitvi17g01082\_t001 |  |  |  |  |  |  |  |  |  |  |  |  |
| 0 | Vvi-Vitvi17g01084\_t001 |  |  |  |  |  |  |  |  |  |  |  |  |
| 0 | Vvi-Vitvi17g01086\_t001 |  |  |  |  |  |  |  |  |  |  |  |  |
| 0 | Vvi-Vitvi17g01087\_t001 |  |  |  |  |  |  |  |  |  |  |  |  |
| 0 | Vvi-Vitvi17g01609\_t001 |  |  |  |  |  |  |  |  |  |  |  |  |
| 0 | Vvi-Vitvi17g01089\_t001 |  |  |  |  |  |  |  |  |  |  |  |  |
| 0 | Vvi-Vitvi17g01092\_t001 |  |  |  |  |  |  |  |  |  |  |  |  |
| 0 | Vvi-Vitvi17g04293\_t001 |  |  |  |  |  |  |  |  |  |  |  |  |
| 0 | Vvi-Vitvi17g04294\_t001 |  |  |  |  |  |  |  |  |  |  |  |  |
| 0 | Vvi-Vitvi17g04295\_t001 |  |  |  |  |  |  |  |  |  |  |  |  |
| 0 | Vvi-Vitvi17g01097\_t001 |  |  |  |  |  |  |  |  |  |  |  |  |
| 0 | Vvi-Vitvi17g01098\_t001 |  |  |  |  |  |  |  |  |  |  |  |  |
| 0 | Vvi-Vitvi17g01610\_t001 |  |  |  |  |  |  |  |  |  |  |  |  |
| 0 | Vvi-Vitvi17g01611\_t001 |  |  |  |  |  |  |  |  |  |  |  |  |
| 0 | Vvi-Vitvi17g01101\_t001 |  |  |  |  |  |  |  |  |  |  |  |  |
| 0 | Vvi-Vitvi17g01102\_t001 |  |  |  |  |  |  |  |  |  |  |  |  |
| 0 | Vvi-Vitvi17g04296\_t001 |  |  |  |  |  |  |  |  |  |  |  |  |
| 0 | Vvi-Vitvi17g01105\_t001 |  |  |  |  |  |  |  |  |  |  |  |  |
| 0 | Vvi-Vitvi17g04297\_t001 |  |  |  |  |  |  |  |  |  |  |  |  |
| 0 | Vvi-Vitvi17g04298\_t001 |  |  |  |  |  |  |  |  |  |  |  |  |
| 0 | Vvi-Vitvi17g01613\_t001 |  |  |  |  |  |  |  |  |  |  |  |  |
| 0 | Vvi-Vitvi17g04299\_t001 |  |  |  |  |  |  |  |  |  |  |  |  |
| 0 | Vvi-Vitvi17g04300\_t001 |  |  |  |  |  |  |  |  |  |  |  |  |
| 0 | Vvi-Vitvi17g04301\_t001 |  |  |  |  |  |  |  |  |  |  |  |  |
| 0 | Vvi-Vitvi17g01617\_t001 |  |  |  |  |  |  |  |  |  |  |  |  |
| 0 | Vvi-Vitvi17g04302\_t001 |  |  |  |  |  |  |  |  |  |  |  |  |
| 0 | Vvi-Vitvi17g04303\_t001 |  |  |  |  |  |  |  |  |  |  |  |  |
| 0 | Vvi-Vitvi17g01620\_t001 |  |  |  |  |  |  |  |  |  |  |  |  |
| 0 | Vvi-Vitvi17g04304\_t001 |  |  |  |  |  |  |  |  |  |  |  |  |
| 0 | Vvi-Vitvi17g01622\_t001 |  |  |  |  |  |  |  |  |  |  |  |  |
| 0 | Vvi-Vitvi17g04305\_t001 |  |  |  |  |  |  |  |  |  |  |  |  |
| 0 | Vvi-Vitvi17g01119\_t001 |  |  |  |  |  |  |  |  |  |  |  |  |
| 0 | Vvi-Vitvi17g01121\_t001 |  |  |  |  |  |  |  |  |  |  |  |  |
| 0 | Vvi-Vitvi17g04306\_t001 |  |  |  |  |  |  |  |  |  |  |  |  |
| 0 | Vvi-Vitvi17g01122\_t001 |  |  |  |  |  |  |  |  |  |  |  |  |
| 0 | Vvi-Vitvi17g04307\_t001 |  |  |  |  |  |  |  |  |  |  |  |  |
| 0 | Vvi-Vitvi17g01124\_t001 |  |  |  |  |  |  |  |  |  |  |  |  |
| 0 | Vvi-Vitvi17g01128\_t001 |  |  |  |  |  |  |  |  |  |  |  |  |
| 0 | Vvi-Vitvi17g04308\_t001 |  |  |  |  |  |  |  |  |  |  |  |  |
| 0 | Vvi-Vitvi17g01628\_t001 |  |  |  |  |  |  |  |  |  |  |  |  |
| 0 | Vvi-Vitvi17g04309\_t001 |  |  |  |  |  |  |  |  |  |  |  |  |
| 0 | Vvi-Vitvi17g04310\_t001 |  |  |  |  |  |  |  |  |  |  |  |  |
| 0 | Vvi-Vitvi17g01138\_t001 |  |  |  |  |  |  |  |  |  |  |  |  |
| 0 | Vvi-Vitvi17g01139\_t001 |  |  |  |  |  |  |  |  |  |  |  |  |
| 1 | Vvi-Vitvi17g04311\_t001 |  | Atr-ERN09447 |  |  |  |  |  |  |  |  |  |  |
| 1 | Vvi-Vitvi17g01140\_t001 |  | \| \| |  |  |  |  |  |  |  |  |  |  |
| 1 | Vvi-Vitvi17g04312\_t001 |  | \| \| |  |  |  |  |  |  |  |  |  |  |
| 1 | Vvi-Vitvi17g04313\_t001 |  | \| \| |  |  |  |  |  |  |  |  |  |  |
| 1 | Vvi-Vitvi17g04314\_t001 |  | \| \| |  |  |  |  |  |  |  |  |  |  |
| 1 | Vvi-Vitvi17g04315\_t001 |  | \| \| |  |  |  |  |  |  |  |  |  |  |
| 1 | Vvi-Vitvi17g01146\_t001 |  | \| \| |  |  |  |  |  |  |  |  |  |  |
| 1 | Vvi-Vitvi17g04316\_t001 |  | \| \| |  |  |  |  |  |  |  |  |  |  |
| 1 | Vvi-Vitvi17g01148\_t001 |  | \| \| |  |  |  |  |  |  |  |  |  |  |
| 1 | Vvi-Vitvi17g04317\_t001 |  | \| \| |  |  |  |  |  |  |  |  |  |  |
| 1 | Vvi-Vitvi17g01633\_t001 |  | \| \| |  |  |  |  |  |  |  |  |  |  |
| 1 | Vvi-Vitvi17g01634\_t001 |  | \| \| |  |  |  |  |  |  |  |  |  |  |
| 1 | Vvi-Vitvi17g01635\_t001 |  | Atr-ERN09423 |  |  |  |  |  |  |  |  |  |  |
| 1 | Vvi-Vitvi17g01155\_t001 |  | \| \| |  |  |  |  |  |  |  |  |  |  |
| 1 | Vvi-Vitvi17g01156\_t001 |  | \| \| |  |  |  |  |  |  |  |  |  |  |
| 1 | Vvi-Vitvi17g01161\_t001 |  | \| \| |  |  |  |  |  |  |  |  |  |  |
| 1 | Vvi-Vitvi17g01162\_t001 |  | Atr-ERN09415 |  |  |  |  |  |  |  |  |  |  |
| 1 | Vvi-Vitvi17g04318\_t001 |  | \| \| |  |  |  |  |  |  |  |  |  |  |
| 1 | Vvi-Vitvi17g01164\_t001 |  | \| \| |  |  |  |  |  |  |  |  |  |  |
| 1 | Vvi-Vitvi17g04319\_t001 |  | \| \| |  |  |  |  |  |  |  |  |  |  |
| 1 | Vvi-Vitvi17g04320\_t001 |  | \| \| |  |  |  |  |  |  |  |  |  |  |
| 1 | Vvi-Vitvi17g04321\_t001 |  | \| \| |  |  |  |  |  |  |  |  |  |  |
| 1 | Vvi-Vitvi17g01165\_t001 |  | \| \| |  |  |  |  |  |  |  |  |  |  |
| 1 | Vvi-Vitvi17g01166\_t001 |  | \| \| |  |  |  |  |  |  |  |  |  |  |
| 1 | Vvi-Vitvi17g04322\_t001 |  | \| \| |  |  |  |  |  |  |  |  |  |  |
| 1 | Vvi-Vitvi17g04323\_t001 |  | \| \| |  |  |  |  |  |  |  |  |  |  |
| 1 | Vvi-Vitvi17g01167\_t001 |  | \| \| |  |  |  |  |  |  |  |  |  |  |
| 1 | Vvi-Vitvi17g01169\_t001 |  | \| \| |  |  |  |  |  |  |  |  |  |  |
| 1 | Vvi-Vitvi17g04324\_t001 |  | \| \| |  |  |  |  |  |  |  |  |  |  |
| 1 | Vvi-Vitvi17g04325\_t001 |  | \| \| |  |  |  |  |  |  |  |  |  |  |
| 1 | Vvi-Vitvi17g04326\_t001 |  | \| \| |  |  |  |  |  |  |  |  |  |  |
| 1 | Vvi-Vitvi17g04327\_t001 |  | \| \| |  |  |  |  |  |  |  |  |  |  |
| 1 | Vvi-Vitvi17g01176\_t001 |  | Atr-ERN09401 |  |  |  |  |  |  |  |  |  |  |
| 1 | Vvi-Vitvi17g04328\_t001 |  | \| \| |  |  |  |  |  |  |  |  |  |  |
| 1 | Vvi-Vitvi17g04329\_t001 |  | \| \| |  |  |  |  |  |  |  |  |  |  |
| 1 | Vvi-Vitvi17g01181\_t001 |  | \| \| |  |  |  |  |  |  |  |  |  |  |
| 1 | Vvi-Vitvi17g01183\_t001 |  | \| \| |  |  |  |  |  |  |  |  |  |  |
| 1 | Vvi-Vitvi17g01184\_t001 |  | \| \| |  |  |  |  |  |  |  |  |  |  |
| 1 | Vvi-Vitvi17g04330\_t001 |  | \| \| |  |  |  |  |  |  |  |  |  |  |
| 1 | Vvi-Vitvi17g01186\_t001 |  | \| \| |  |  |  |  |  |  |  |  |  |  |
| 1 | Vvi-Vitvi17g01643\_t001 |  | \| \| |  |  |  |  |  |  |  |  |  |  |
| 1 | Vvi-Vitvi17g04331\_t001 |  | \| \| |  |  |  |  |  |  |  |  |  |  |
| 1 | Vvi-Vitvi17g04332\_t001 |  | \| \| |  |  |  |  |  |  |  |  |  |  |
| 1 | Vvi-Vitvi17g01644\_t001 |  | Atr-ERN09393 |  |  |  |  |  |  |  |  |  |  |
| 1 | Vvi-Vitvi17g04333\_t001 |  | \| \| |  |  |  |  |  |  |  |  |  |  |
| 1 | Vvi-Vitvi17g01190\_t001 |  | Atr-ERN09383 |  |  |  |  |  |  |  |  |  |  |
| 1 | Vvi-Vitvi17g04334\_t001 |  | \| \| |  |  |  |  |  |  |  |  |  |  |
| 1 | Vvi-Vitvi17g01646\_t001 |  | \| \| |  |  |  |  |  |  |  |  |  |  |
| 1 | Vvi-Vitvi17g04335\_t001 |  | \| \| |  |  |  |  |  |  |  |  |  |  |
| 1 | Vvi-Vitvi17g01647\_t001 |  | Atr-ERN09382 |  |  |  |  |  |  |  |  |  |  |
| 0 | Vvi-Vitvi17g04336\_t001 |  |  |  |  |  |  |  |  |  |  |  |  |
| 0 | Vvi-Vitvi17g01197\_t001 |  |  |  |  |  |  |  |  |  |  |  |  |
| 0 | Vvi-Vitvi17g01198\_t001 |  |  |  |  |  |  |  |  |  |  |  |  |
| 0 | Vvi-Vitvi17g04337\_t001 |  |  |  |  |  |  |  |  |  |  |  |  |
| 0 | Vvi-Vitvi17g04338\_t001 |  |  |  |  |  |  |  |  |  |  |  |  |
| 0 | Vvi-Vitvi17g04339\_t001 |  |  |  |  |  |  |  |  |  |  |  |  |
| 0 | Vvi-Vitvi17g01650\_t001 |  |  |  |  |  |  |  |  |  |  |  |  |
| 0 | Vvi-Vitvi17g01651\_t001 |  |  |  |  |  |  |  |  |  |  |  |  |
| 0 | Vvi-Vitvi17g04340\_t001 |  |  |  |  |  |  |  |  |  |  |  |  |
| 0 | Vvi-Vitvi17g01202\_t001 |  |  |  |  |  |  |  |  |  |  |  |  |
| 0 | Vvi-Vitvi17g04341\_t001 |  |  |  |  |  |  |  |  |  |  |  |  |
| 0 | Vvi-Vitvi17g04342\_t001 |  |  |  |  |  |  |  |  |  |  |  |  |
| 0 | Vvi-Vitvi17g04343\_t001 |  |  |  |  |  |  |  |  |  |  |  |  |
| 0 | Vvi-Vitvi17g01203\_t001 |  |  |  |  |  |  |  |  |  |  |  |  |
| 0 | Vvi-Vitvi17g01204\_t001 |  |  |  |  |  |  |  |  |  |  |  |  |
| 0 | Vvi-Vitvi17g04344\_t001 |  |  |  |  |  |  |  |  |  |  |  |  |
| 0 | Vvi-Vitvi17g01206\_t001 |  |  |  |  |  |  |  |  |  |  |  |  |
| 0 | Vvi-Vitvi17g01207\_t001 |  |  |  |  |  |  |  |  |  |  |  |  |
| 0 | Vvi-Vitvi17g04345\_t001 |  |  |  |  |  |  |  |  |  |  |  |  |
| 0 | Vvi-Vitvi17g01209\_t001 |  |  |  |  |  |  |  |  |  |  |  |  |
| 0 | Vvi-Vitvi17g04346\_t001 |  |  |  |  |  |  |  |  |  |  |  |  |
| 0 | Vvi-Vitvi17g04347\_t001 |  |  |  |  |  |  |  |  |  |  |  |  |
| 0 | Vvi-Vitvi17g04348\_t001 |  |  |  |  |  |  |  |  |  |  |  |  |
| 0 | Vvi-Vitvi17g04349\_t001 |  |  |  |  |  |  |  |  |  |  |  |  |
| 0 | Vvi-Vitvi17g04350\_t001 |  |  |  |  |  |  |  |  |  |  |  |  |
| 0 | Vvi-Vitvi17g04351\_t001 |  |  |  |  |  |  |  |  |  |  |  |  |
| 0 | Vvi-Vitvi17g04352\_t001 |  |  |  |  |  |  |  |  |  |  |  |  |
| 0 | Vvi-Vitvi17g04353\_t001 |  |  |  |  |  |  |  |  |  |  |  |  |
| 0 | Vvi-Vitvi17g04354\_t001 |  |  |  |  |  |  |  |  |  |  |  |  |
| 0 | Vvi-Vitvi17g04355\_t001 |  |  |  |  |  |  |  |  |  |  |  |  |
| 0 | Vvi-Vitvi17g01217\_t001 |  |  |  |  |  |  |  |  |  |  |  |  |
| 0 | Vvi-Vitvi17g01218\_t001 |  |  |  |  |  |  |  |  |  |  |  |  |
| 0 | Vvi-Vitvi17g01220\_t001 |  |  |  |  |  |  |  |  |  |  |  |  |
| 0 | Vvi-Vitvi17g04356\_t001 |  |  |  |  |  |  |  |  |  |  |  |  |
| 0 | Vvi-Vitvi17g01221\_t001 |  |  |  |  |  |  |  |  |  |  |  |  |
| 0 | Vvi-Vitvi17g01223\_t001 |  |  |  |  |  |  |  |  |  |  |  |  |
| 0 | Vvi-Vitvi17g01224\_t001 |  |  |  |  |  |  |  |  |  |  |  |  |
| 0 | Vvi-Vitvi17g04357\_t001 |  |  |  |  |  |  |  |  |  |  |  |  |
| 0 | Vvi-Vitvi17g04358\_t001 |  |  |  |  |  |  |  |  |  |  |  |  |
| 0 | Vvi-Vitvi17g01227\_t001 |  |  |  |  |  |  |  |  |  |  |  |  |
| 0 | Vvi-Vitvi17g01228\_t001 |  |  |  |  |  |  |  |  |  |  |  |  |
| 0 | Vvi-Vitvi17g01229\_t001 |  |  |  |  |  |  |  |  |  |  |  |  |
| 0 | Vvi-Vitvi17g01232\_t001 |  |  |  |  |  |  |  |  |  |  |  |  |
| 0 | Vvi-Vitvi17g01656\_t001 |  |  |  |  |  |  |  |  |  |  |  |  |
| 0 | Vvi-Vitvi17g04359\_t001 |  |  |  |  |  |  |  |  |  |  |  |  |
| 0 | Vvi-Vitvi17g01233\_t001 |  |  |  |  |  |  |  |  |  |  |  |  |
| 0 | Vvi-Vitvi17g01234\_t001 |  |  |  |  |  |  |  |  |  |  |  |  |
| 0 | Vvi-Vitvi17g04360\_t001 |  |  |  |  |  |  |  |  |  |  |  |  |
| 0 | Vvi-Vitvi17g01236\_t002 |  |  |  |  |  |  |  |  |  |  |  |  |
| 0 | Vvi-Vitvi17g04361\_t001 |  |  |  |  |  |  |  |  |  |  |  |  |
| 0 | Vvi-Vitvi17g01658\_t001 |  |  |  |  |  |  |  |  |  |  |  |  |
| 0 | Vvi-Vitvi17g04362\_t001 |  |  |  |  |  |  |  |  |  |  |  |  |
| 0 | Vvi-Vitvi17g04363\_t001 |  |  |  |  |  |  |  |  |  |  |  |  |
| 0 | Vvi-Vitvi17g04364\_t001 |  |  |  |  |  |  |  |  |  |  |  |  |
| 0 | Vvi-Vitvi17g04365\_t001 |  |  |  |  |  |  |  |  |  |  |  |  |
| 0 | Vvi-Vitvi17g04366\_t001 |  |  |  |  |  |  |  |  |  |  |  |  |
| 0 | Vvi-Vitvi17g04367\_t001 |  |  |  |  |  |  |  |  |  |  |  |  |
| 0 | Vvi-Vitvi17g01250\_t001 |  |  |  |  |  |  |  |  |  |  |  |  |
| 0 | Vvi-Vitvi17g04368\_t001 |  |  |  |  |  |  |  |  |  |  |  |  |
| 0 | Vvi-Vitvi17g01251\_t001 |  |  |  |  |  |  |  |  |  |  |  |  |
| 0 | Vvi-Vitvi17g04369\_t001 |  |  |  |  |  |  |  |  |  |  |  |  |
| 0 | Vvi-Vitvi17g01254\_t001 |  |  |  |  |  |  |  |  |  |  |  |  |
| 0 | Vvi-Vitvi17g04370\_t001 |  |  |  |  |  |  |  |  |  |  |  |  |
| 0 | Vvi-Vitvi17g04371\_t001 |  |  |  |  |  |  |  |  |  |  |  |  |
| 0 | Vvi-Vitvi17g04372\_t001 |  |  |  |  |  |  |  |  |  |  |  |  |
| 0 | Vvi-Vitvi17g04373\_t001 |  |  |  |  |  |  |  |  |  |  |  |  |
| 0 | Vvi-Vitvi17g04374\_t001 |  |  |  |  |  |  |  |  |  |  |  |  |
| 0 | Vvi-Vitvi17g01264\_t001 |  |  |  |  |  |  |  |  |  |  |  |  |
| 0 | Vvi-Vitvi17g01265\_t001 |  |  |  |  |  |  |  |  |  |  |  |  |
| 0 | Vvi-Vitvi17g01266\_t001 |  |  |  |  |  |  |  |  |  |  |  |  |
| 0 | Vvi-Vitvi17g01268\_t001 |  |  |  |  |  |  |  |  |  |  |  |  |
| 0 | Vvi-Vitvi17g01660\_t001 |  |  |  |  |  |  |  |  |  |  |  |  |
| 0 | Vvi-Vitvi17g01269\_t001 |  |  |  |  |  |  |  |  |  |  |  |  |
| 0 | Vvi-Vitvi17g01661\_t001 |  |  |  |  |  |  |  |  |  |  |  |  |
| 0 | Vvi-Vitvi17g01662\_t001 |  |  |  |  |  |  |  |  |  |  |  |  |
| 0 | Vvi-Vitvi17g01270\_t001 |  |  |  |  |  |  |  |  |  |  |  |  |
| 0 | Vvi-Vitvi17g01271\_t001 |  |  |  |  |  |  |  |  |  |  |  |  |
| 0 | Vvi-Vitvi17g04375\_t001 |  |  |  |  |  |  |  |  |  |  |  |  |
| 0 | Vvi-Vitvi17g04376\_t001 |  |  |  |  |  |  |  |  |  |  |  |  |
| 0 | Vvi-Vitvi17g04377\_t001 |  |  |  |  |  |  |  |  |  |  |  |  |
| 0 | Vvi-Vitvi17g04378\_t001 |  |  |  |  |  |  |  |  |  |  |  |  |
| 0 | Vvi-Vitvi17g04379\_t001 |  |  |  |  |  |  |  |  |  |  |  |  |
| 0 | Vvi-Vitvi17g04380\_t001 |  |  |  |  |  |  |  |  |  |  |  |  |
| 0 | Vvi-Vitvi17g04381\_t001 |  |  |  |  |  |  |  |  |  |  |  |  |
| 0 | Vvi-Vitvi17g04382\_t001 |  |  |  |  |  |  |  |  |  |  |  |  |
| 0 | Vvi-Vitvi17g04383\_t001 |  |  |  |  |  |  |  |  |  |  |  |  |
| 0 | Vvi-Vitvi17g04384\_t001 |  |  |  |  |  |  |  |  |  |  |  |  |
